# A Software Deep Packet Inspection System for Network Traffic Analysis and Anomaly Detection

**DOI:** 10.3390/s20061637

**Published:** 2020-03-14

**Authors:** Wenguang Song, Mykola Beshley, Krzysztof Przystupa, Halyna Beshley, Orest Kochan, Andrii Pryslupskyi, Daniel Pieniak, Jun Su

**Affiliations:** 1 School of Computer Science, Yangtze University, Jingzhou 434023, China; wenguang_song@yangtzeu.edu.cn; 2 Department of telecommunications, Lviv Polytechnic National University, Bandery 12, 79013 Lviv, Ukraine; mykola.i.beshlei@lpnu.ua (M.B.); halink@ukr.net (H.B.); orestvk@gmail.com (O.K.); pr.andrii@gmail.com (A.P.); 3 Department of Automation, Lublin University of Technology, Nadbystrzycka 36, 20-618 Lublin, Poland; 4 Department of Mechanics and Machine Building, University of Economics and Innovations in Lublin, Projektowa 4, 20-209 Lublin, Poland; daniel.pieniak@wsei.lublin.pl; 5 School of Computer Science, Hubei University of Technology, Wuhan 430068, China; sjhosix@gmail.com

**Keywords:** IoT, WSN, network anomaly, Hurst parameter, DPI, intrusion detection

## Abstract

In this paper, to solve the problem of detecting network anomalies, a method of forming a set of informative features formalizing the normal and anomalous behavior of the system on the basis of evaluating the Hurst (H) parameter of the network traffic has been proposed. Criteria to detect and prevent various types of network anomalies using the Three Sigma Rule and Hurst parameter have been defined. A rescaled range (RS) method to evaluate the Hurst parameter has been chosen. The practical value of the proposed method is conditioned by a set of the following factors: low time spent on calculations, short time required for monitoring, the possibility of self-training, as well as the possibility of observing a wide range of traffic types. For new DPI (Deep Packet Inspection) system implementation, algorithms for analyzing and captured traffic with protocol detection and determining statistical load parameters have been developed. In addition, algorithms that are responsible for flow regulation to ensure the QoS (Quality of Services) based on the conducted static analysis of flows and the proposed method of detection of anomalies using the parameter Hurst have been developed. We compared the proposed software DPI system with the existing SolarWinds Deep Packet Inspection for the possibility of network traffic anomaly detection and prevention. The created software components of the proposed DPI system increase the efficiency of using standard intrusion detection and prevention systems by identifying and taking into account new non-standard factors and dependencies. The use of the developed system in the IoT communication infrastructure will increase the level of information security and significantly reduce the risks of its loss.

## 1. Introduction

One of the manifestations of the society informatization process is the large-scale development of network services, such as Internet of Things (IoT) services, Wireless Sensor Network (WSN) services, Cloud services, Wireless Sensor Multimedia Networks (WSMN) services, etc. [1,2,3]. Administrators of information systems, providing the services, face the task of ensuring the manageability and accountability of these systems, data integrity, availability, and confidentiality. They also ensure the system’s regular functioning and exclude as much as possible incidents of non-standard functioning and network anomalies. 

With the increasing use of IoT and WSMN infrastructure, threats and attacks in existing corporate network domains are growing proportionally in this infrastructure. The detection of attacks and anomalies in the Internet of Things (IoT) infrastructure is a growing concern for IoT [4,5].

An anomaly is a deviation from generally accepted norms. Therefore, everything that deviates from right or normal is called anomalous. Network anomalies are defined as deviations in the use of network resources that are accessed through web services and network applications. Network anomalies have different causes and may be related to hackers, incompetent users, hardware malfunctions, and software defects. There are visible anomalies that appear directly in the incorrect operation of the information computer system. The anomalies may not have visible signs, but they can cause failures after a long time [6].

Anomaly detection is now one of the fastest growing areas of cybersecurity [7]. This is due to the fact that anomalies are in most cases the initial stage of network attacks, which can have both negative intangible effects and financial losses for organizations with substantial representation in cyberspace. Typically, such anomalies are the result of intelligence or a "power-down" to further exploit detected security problems for commercial gain. 

Understanding the nature of network traffic anomalies is an important task. Whether or not the anomalies are malicious, it is important to analyze them for two reasons:

- Anomalies can cause network congestion and increase router resource utilization, making their detection critical.
- Some anomalies do not necessarily affect the network, but they can have a serious impact on a client or end user.

A significant problem with anomaly detection is that the forms of anomalies can change depending on the cause: from DoS attacks (denial of service) to incorrect configurations of the router. Denial of Service, Data Type Probing, Malicious Control, Malicious Operation, Scan, Spying, and Wrong Setup are such attacks and anomalies that can lead to failure of not only the IoT system, but across the entire network infrastructure [8].

Detection and classification of anomalies assumes a continuous process of monitoring of events in computer systems and networks; in this connection, processing big volumes of the data generated by these sources is required. Using a deep packet inspection that combines the functionality of an intrusion detection system (IDS) and an intrusion prevention system (IPS) is a good solution to detect network anomalies and attacks [9]. Recent research shows that in the era of data encryption, the system can still classify encrypted traffic, ensuring data security [10]. DPI-based solutions provide a complete picture of network usage, identify subscribers that consume large amounts of traffic, and effectively manage traffic in real time to help create or optimize service offerings, improve service quality, manage service policies, and protect the network and its users [11,12,13]. Typically, DPI systems are installed at the most loaded segments of the network (trunk links and connections to higher networks) and at points where service management is required. By examining the packets passing through it (including the data field), DPI determines which type of application a particular session belongs to and applies the rules defined by the administrator. In this way, the administrator can block, restrict, prioritize, or redirect traffic to other systems that meet the specified conditions. DPI uses rules based on signature analysis as well as heuristic and statistical technologies to determine the protocols, so the rules specified by the administrator will be applied based on the traffic being transmitted, even if the traffic is transmitted on ports that are non-standard for the application used [14]. The signature-based analysis methods used in modern intrusion detection systems are designed to detect known and accurately described types of attacks, and they are unable to detect their modifications or new types, which makes the use of such systems ineffective. The existing solutions for detecting network anomalies have so far prevented the development of a single universal mechanism to detect previously unknown types of attacks. DPIs are commercial software products that allow analyzing traffic for anomalies and threats in real time. The limiting factors of using such systems are their high cost and closed architecture, which makes it difficult to adapt them to the organization infrastructure.

Despite the large amount of literature that describes basic network traffic characteristics, traffic anomalies remain poorly understood [15,16,17,18]. There are many reasons for this. They require a sophisticated monitoring infrastructure to identify anomalies, as well as tools to process measurements fast enough to detect anomalies in real time. Another reason is that the nature of network traffic is multidimensional and multiservice, which prevents useful anomaly information from being obtained from traffic statistics.

The actual task at the moment is to find more effective universal methods of network anomalies detection, which are the result of technical failures or unauthorized influence. The main requirement for these methods is the possibility of detecting arbitrary types of anomalies, including those distributed over time. Statistical studies of network traffic show the presence of self-similarity properties, as well as the variability of these characteristics when anomalies appear in the network, which allows using fractal analysis methods to detect attacks [19,20].

For this reason, the purpose of this paper is to develop effective algorithms for analysis of the most used information protocols and to create a software DPI system that collects statistical data for the further control of traffic in the network operator on the basis of the proposed Hurst parameter estimation method for detecting and preventing network anomalies.

Thus, the proposed DPI system allows solving several important tasks at once: from optimizing bandwidth and prioritizing traffic to the behavioral evaluation of subscribers and protecting networks and sites from all kinds of attacks

This paper is organized as follows. Section 2 describes the related research work on network anomaly detection methods, DPI systems, network traffic analysis, and anomaly detection in IoT and WSN. Then, Section 3 introduces the proposed method for network traffic anomaly detection and prevention. Section 4 describes the development of the software DPI system for network traffic analysis and anomaly detection. Section 5 introduces the test bed for network traffic analysis and anomaly detection, and it also includes the results and discussion. Finally, Section 6 concludes this work.

## 2. Related Work

### 2.1. Background on Network Anomaly Detection Methods

In this part, we illustrate the research status of development of the anomaly detection methods for the information network. 

Three commonly used categories of anomaly detection techniques are listed below:

*Unsupervised anomaly detection technique.* Systems using such techniques do not require pre-prepared data and are, therefore, the most widely used. It is assumed that normal data in a dataset is much more common than anomalous data. If the assumption is incorrect, systems built using such techniques suffer from frequent false positives [21].

*Supervised anomaly detection techniques.* This technique assumes that there are two classes of entities for normal and abnormal behavior. Normally, a model is constructed for the normal and anomaly classes, after which the data not previously studied are compared to both classes to find out the one to which one it belongs [22].

*A semi-supervised anomaly detection technique.* The technique implies that the data studied are only available for the "norm" class. Considering that it is much easier to construct only a model of normal behavior (since it is impossible to foresee all possible anomalies), this technique is more widely applicable than the controlled method [23].

At the moment, there are quite a few methods to detect network traffic anomalies. They can be grouped as follows:

*Signature methods.* The most commonly used group of methods, the essence of which consists of compiling some alphabet from the events observed in the system and describing a set of attack signatures in the form of regular expressions (in the general case) in the built alphabet. As a rule, signature methods work at the lowest level of abstraction and analyze data that are directly transmitted over the network, system call parameters, and log file entries. The principle of this method’s operation is described in the article. Signature methods are notable for the fact that hardware accelerators are well used for them, but the method is not adaptive [24].

*Neural networks using the knowledge base.* Neural networks learn to detect anomalies for a period of time when all behavior is considered normal. After training, the neural network is started in recognition mode. In a situation where the input stream fails to recognize normal behavior, an attack is recorded. Thus, by combining two different neural networks, you can identify and recognize computer attacks with a fairly high degree of accuracy. The main advantages of using approaches based on neural networks are the ability to adapt to dynamic conditions and the performance rates, which are especially important when the system is operating in real time [25]. The disadvantage is that the development of quality knowledge bases requires a lot of effort and time. Such methods are unable to detect a rare or unknown anomaly.

*Immune networks.* The detection of anomalies is one of the possible applications of immune methods. Since the number of examples of normal behavior usually exceeds the number of attack examples by orders of magnitude, the use of immune networks for detecting anomalies is more computationally complicated [26]. 

*Expert systems.* Information about normal behavior is presented in such systems in the form of rules and monitored behavior in the form of facts. Based on the facts and rules, a decision is made on whether the monitored behavior is "normal" or whether there is an anomaly [10]. The main disadvantage of such systems is their high computational complexity (in the general case), in particular at the detection of anomalies [27].

*Cluster analysis*. The essence of this group of methods is to divide the set of observable vectors and system properties into clusters, among which are allocated clusters of normal behavior. Each specific method of cluster analysis uses its own metrics, which allows assessing whether the observed vector of system properties belongs to one of the clusters or is outside of the known clusters [9]. Most of the methods based on clustering have been proposed for processing only continuous attributes. The assumption is that large clusters are the norm, and small clusters are an anomaly. If it is not, then the work of the method is difficult. Using an inappropriate measure of proximity of objects affects the frequency of false alarms [28].

*Statistical analysis*. This group of methods is based on building a statistical profile of the system’s behavior during a certain period of "learning", in which the system’s behavior is considered normal. For each parameter of the system functioning, an interval of acceptable values is built using some known distribution law. Further, in the detection mode, the system evaluates deviations of observed values from the values obtained during the training. If deviations exceed some specified values, an anomaly (attack) is recorded. Statistical analysis is characterized by a high level of false alarms when used in local networks, where the behavior of objects has no smooth, averaged character. In addition, this method is only stable within a particular system, i.e., the constructed statistical profiles cannot be used on other similar systems. The advantage of this approach is that it does not require prior knowledge about the properties of anomalies and can therefore be effective with unknown anomalies and even with changes in existing known anomalies. One statistical method of detection is a method based on fractal analysis [29,30,31,32].

The use of the described types of methods and systems of anomaly detection allows strengthening security policies and bringing more flexibility in the process of network resources exploitation. However, any system has certain advantages as well as some disadvantages. At the analysis of these means, it appears that the majority of them carry out only one or several specific functions that cannot provide a certain degree of complexity of information protection. As a result of the carried analysis of methods and systems for detecting anomalies, it is allocated that only due to a combination of several methods and systems is it possible to reach effective protection and to provide counteraction to threats.

### 2.2. Background on DPI System

At the moment, there are a large number of intrusion detection systems. Commercial and open-source systems can be distinguished for classification by the distribution method. Since the algorithms of anomaly detection engines in commercial intrusion detection systems are in the vast majority of cases an object of commercial secret, it is logical to describe the most famous open-source projects—Snort, SolarWinds Network Performance Monitor and Suricata—to illustrate the principles of work [33,34,35].

Snort is an open source free network intrusion, prevention, and detection system (IPS) with the ability to perform packet registration and real-time traffic analysis in IP networks. So, the packet received by Snort goes through decoders and preprocessors, and then it gets to the detector that applies the rules. The task of the decoder is to separate the network and transport layer data (IP (Internet Protocol), TCP (Transmission Control Protocol), UDP (User Datagram Protocol)) from the channel layer protocols (Ethernet, 802.11, Token Ring...). The preprocessor task is to prepare data for rule application. As a result, before entering the detector, prepared packets are formed, to which the detector begins to apply the rules [36]. The rules themselves consist of a description of traffic, the required signature, a description of the threat, and a description of the response to detection. Snort is a software product, and although it has a proven track record, it still has some shortcomings. Similar to Snort, Suricata is a free SWS (Software System) and PSB (Program Specification Block) with open source code. It was founded by developers who worked on the IPS version of Snort [37]. The main differences between Suricata and Snort include the ability to use the GPU (graphics processor) in IDS (Intrusion Detection System) mode, more advanced system IPS, and multitasking. As a consequence, Suricata has high performance, allowing to process traffic up to 10 Gbit on conventional equipment, and much more, including full support for the format of rules Snort. Similar to Snort, Suricata consists of several modules: capture, collection, decoding, detection, and output [38]. The most important feature of Suricata is that in addition to its unique developments, it uses almost everything that is already implemented for Snort.

With the advent of encrypted protocols, the task of identifying network protocols became more complex, which led to new developments. Work [39] considers the development and realization of nDPI, which are libraries with an open source code for classification of protocols. This approach uses package header and payload. The testing of the nDPI system in practice has confirmed the high efficiency and accuracy of protocol detection. Another paper [40] proposes a practical approach for improving the efficiency of traditional traffic classification methods by consistently passing the rapid classification stages (based on ports and machine learning). Although the proposed method reduces false positives and is more accurate, it requires significant time and resources when developing a DPI system. The authors of work [41] propose to use deep learning (DL) techniques to develop practical classifiers of mobile traffic that are capable of handling encrypted traffic. The basic problems regarding the realization of such classifiers are considered and the efficiency of use of the offered classifier is shown.

With the growing spread of computer networks and services that depend on them, the problem of detecting abnormal network activity becomes more and more acute. Network anomalies and their detection techniques have an established classification. Anomaly detection methods also have a sufficiently detailed classification, which helps to determine the method used by a modern intrusion detection system.

### 2.3. Related Research on Network Traffic Analysis and Anomaly Detection in IoT and WSN

WSNs and IoT have become very popular recently [42,43]. These networks, consisting of many miniature nodes equipped with a low-power transceiver, microprocessor, and sensor, can connect global computer networks with the physical world [44,45,46]. The concept of wireless sensor networks has attracted the attention of many scientists, research institutes, and commercial organizations, which has provided a large flow of scientific work on this topic [47,48,49,50,51,52,53]. The integration of low-power wireless networking technologies such as (WSNs) and low-cost cameras and microphones has allowed the development of so-called WMSNs (Wireless Multimedia Sensor Networks) [54]. 

The potential and feasibility of graph-based deep learning for detecting anomalies in these WSNs are also explored. Finally, some remarks on modeling anomaly detection methods, using appropriate datasets for validation purposes, and interpreting complex machine learning models are given. Unfortunately, such methods are unable to detect a rare or unknown network anomaly [55]. 

The authors [56] propose a new approach for the automatic detection of anomalies in heterogeneous sensor networks, based on the analysis of data on the boundaries of communication with the analysis of cloud data. This approach uses a fully unmanaged artificial neural network algorithm, while cloud data analysis uses a multi-parameter editing distance algorithm. The experimental evaluation of the proposed method is carried out using edge and cloud analysis on real data, which were obtained in the internal environment of the building, and then distorted with a number of synthetic violations. The obtained results show that the proposed method can self-adapt to environmental changes and correctly detect anomalies. The authors show how a combination of edge and cloud computing can mitigate the disadvantages of pure edge analysis or pure cloud solutions.

In paper [57], the authors presents a literature survey of the work done in the field in recent years focusing primarily on machine learning techniques to network anomaly detection. Major research gaps regarding the practical feasibility of these schemes are also identified from surveyed work in Industrial Wireless Sensor Networks (IWSN), and critical water infrastructure is discussed as a use case.

In order to more effectively detect new attacks, a model of anomaly detection using the Hurst exponent vector and the multifractal spectrum is proposed in work [58]. It is shown that a multifractal analysis shows sensitivity to any deviation of network traffic properties resulting from anomalies. Proposed traffic analysis methods can be ideal for protecting critical data and maintaining the continuity of Internet services, including the IoT. The disadvantage of the proposed method for detecting anomalies requires a significant monitoring interval, preventing a rapid response to anomalies.

The authors [59] explore the use of big data and machine learning to identify abnormal actions that can occur in a smart home environment. The Hidden Mark Model (HMM) learns how to handle network level sensor data from a multi-sensor test bed with intelligent devices. The generated HMM shows 97% accuracy when identifying potential anomalies that indicate an attack.

The survey [60] gives a brief idea about challenges in DPI and some of the design objectives and then briefly explains different matching algorithms and their limitations. At the end, some of the most popular techniques using DPI are outlined.

The paper [61] suggests a new model to improve the performance of intrusion detection systems by using in/out-based attributes of records. It takes comparatively less time and has better accuracy than the existing classifiers.

In the course of the review of the work, the existing methods of anomaly detection and tools for this purpose were studied. It turned out that despite the large number of existing research in this field, the topic has not lost its relevance, and the existing solutions still have a number of significant shortcomings. In addition, a number of facts and regularities were found in the course of the study that allowed the authors of the work to offer a new method based on the calculation of the Hurst parameter as a digital value to indicate the normality or anomaly of network traffic.

## 3. Description of the Proposed Method for Network Traffic Anomaly Detection and Prevention

As a solution to the problem of network anomalies detection, a new method is proposed based on the algorithm developed by the authors below.

The approach implies the development of a software–hardware complex (SHC) for detecting network anomalies. Under SHC, we mean the installation of the software product developed by the authors, which performs the functions of DPI on available hardware platforms (servers). In addition, modern technologies already allow making full-featured DPI in the form of a special router [62]. 

Key steps in using the method (approach):

1. Installing the device (SHC) in the enterprise network;
2. Capturing of subscriber traffic and processing using an algorithm;
3. Reaction to the detected anomaly (depends on the installation scheme).

A summary principle of the algorithm’s operation is described below.

In order to detect an anomaly, it is proposed to use a table of reference values for each new subscriber who appears in the network. The algorithm has two stages: the Training stage and the Detection stage. The algorithm also has three main components: Collector, Analyzer, and Regulator. The list of objects for which the reference values are calculated is the subject of discussion, but it is obvious that the key objects are the values of the transport and network level headers. In a simple interpretation, data obtained during the training phase will be compared with data obtained during the detection phase after its completion. If the training stage is final (authors proposes to set this training to 1 minute from the moment the subscriber appears in the field of view of the system), the detection stage is not present, and it is a cyclic repetition of the calculation of the value for the time window of monitoring and comparison with the reference values.

The authors of this paper proposed using the Hurst parameter *(H)* as an anomaly criterion. The authors assumed that by making a number of control measurements and filling out the subscriber’s table, it is possible in further monitorings to draw conclusions about the normality or anomaly of traffic based on the remoteness of the actual values received from the reference. The *H* parameter, which is called the Hurst parameter, is a measure of self-similarity or a measure of the long-term dependence of the stochastic process. A value H = 0.5 indicates that there is no long-term dependence. The closer the *H* value is to 1, the higher the degree of stability of the long-term relationship. This is true for large samples, while in this work, it is suggested to use very small samples, which is also the author’s idea.

There are a number of methods to determine the Hurst parameter: *R/S*-analysis (normalized scale method), the change in time of dispersion of an aggregated series, calculation using wavelet analysis, and fractality index determination. Taking into account the needs of the authors of this paper (the need to obtain a certain final value), *R/S* analysis of the time series was chosen. In spite of the fact that R/S-analysis gives only an approximate value of the Hurst index [63], the decisive factor was the simplicity of the calculations. The wavelet analysis was also considered, but it was discarded due to the fact that the complexity of calculations and the sample volume necessary for the calculation of scaling factors are excessive for the method proposed in this paper.

After receiving the values and writing them to the table, the system will calculate the average value for each group of values by intervals, as well as the standard deviation *(S_N_).* Based on these data, the system will build an interval according to the Three Sigma rule, and in the case of the proposed method, when the average value of the entire sample is unknown. When the Three Sigma rule is applied, provided the normal distribution, all values with a probability of more than 99% will fall [64]. This is necessary to estimate the level of anomaly depending on the hitting of the value obtained at the stage of detection, within a range of *(-3 S_N_; +3 S_N_).*

The main idea is that the packets from each subscriber of the internal network run through two stages: training and discovery. The training phase lasts for a specified period of time, and after its completion, the system will have a table of values for this subscriber. Going to the detection stage, the system will compare the real-time calculations for certain intervals with the value in the table and make conclusions about the presence of anomaly activity. A table is maintained for each subscriber, and the training and detection stages are different for each subscriber.

### 3.1. Block Diagram of the Network Anomaly Detection Algorithm Based On Hurst Parameter Estimation by R/S Method

The collector captures the traffic and obtains digital values from the packet headers per time unit of monitoring. By monitoring unit, we mean a group of values for a certain time interval. The collector is also engaged in sorting traffic by subscribers and transmitting values for further processing to the analyzer.

The analyzer, in its turn, applies mathematical formulas to the values received from the collector and must decide what to do with the traffic further. To do it, it needs to understand whether the subscriber is new or has been in the system’s sight before. If the subscriber is new, the system moves on to the training stage, i.e., a new blank table of reference values is started, and its filling starts. If the subscriber is already in the system base, the analyzer must understand what stage he is at. If the subscriber is in the training stage, it is necessary to continue writing to the existing table.

The time mark in a certain cell in the table will allow the analyzer to understand how long the training stage has been started. If the subscriber is at the training stage, this is where it ends. If a subscriber is at the detection stage, the time stamp is forwarded to the regulator. The regulator compares the values obtained from the analyzer with the values from tables (obtained during the training stage) and makes conclusions about the presence or absence of an anomaly. If an anomaly is detected, the action is applied. An action may be some kind of a sanction (for example, the limitation of ICMP traffic for a subscriber) or passive behavior (notification of the system administrator about a potential problem).

**Figure 1:**
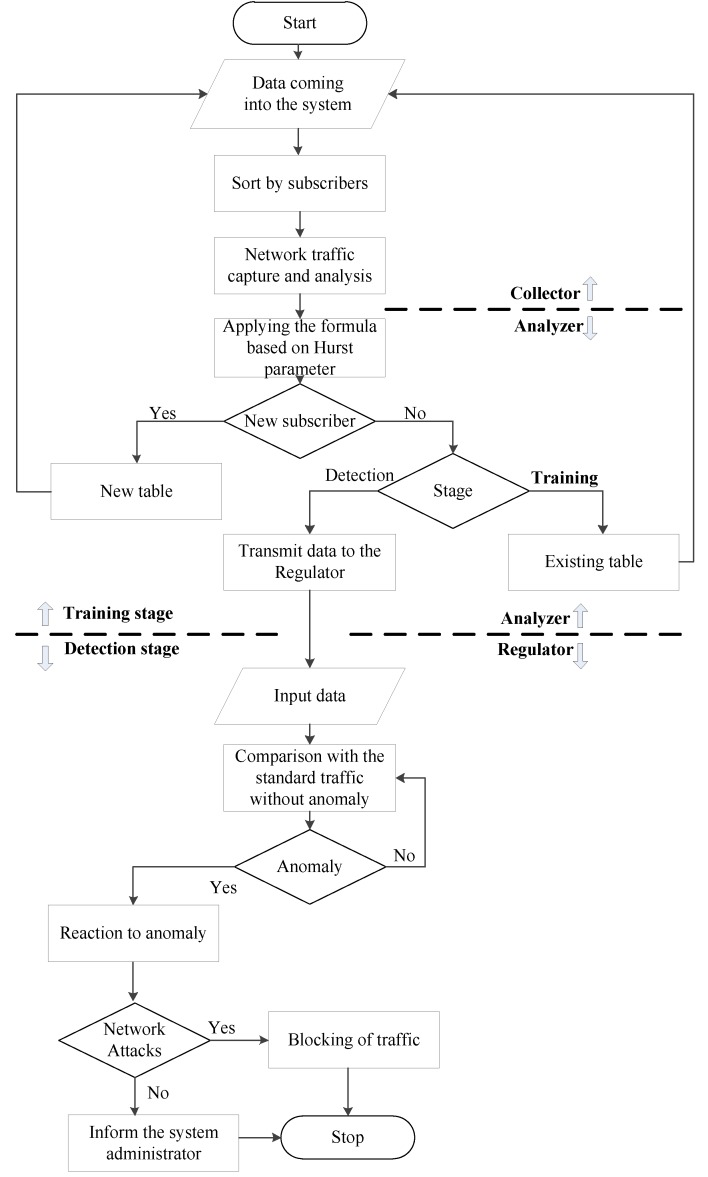
Block diagram of the anomaly detection algorithm.

The below shows a block diagram of the algorithm (Figure 1).

As discussed above, it is proposed to use R/S analysis to calculate the Hurst parameter as a method for obtaining a numerical value (which will be written to Table 1).

Its essence is as follows.

First, it determines the average value of the traffic packets intensity $X_{k}\left(k=1\ldots N\right)$, in which *N* stands for the size of the sample:

(1) $$M_{N}=\frac{1}{N}\sum_{k=1}^{N}X_{k}.$$

The dispersion of traffic packets intensity is determined $X_{k}\left(k=1\ldots N\right)$:

(2) $$S_{N}^{2}=\frac{1}{N}\sum_{k=1}^{N}{\left(X_{k}-M_{N}\right)}^{2}.$$

Standard deviation is defined as follows:

(3) $$S_{N}^{}=\sqrt{\frac{1}{N}\sum_{k=1}^{N}{\left(X_{k}-M_{N}\right)}^{2}}.$$

If a small sample is selected N < 50, the Bessel correction is entered, and the standard deviation is defined as follows:

(4) $$S_{N}^{}=\sqrt{\frac{1}{N-1}\sum_{k=1}^{N}{\left(X_{k}-M_{N}\right)}^{2}}.$$

For estimation of the range of the traffic packets intensity values, it is suggested to use the integral deviation, which is an interval determination of the deviation of the sum of the network traffic intensity values j from the average intensity values j.

The integral deviation is determined:

(5) $$D_{j}=\sum_{k=1}^{j}X_{k}-jM,j\in \left[1;N\right].$$

Accordingly, an array of data is formed, for which the range is determined as the difference between the maximum and minimum values of the integral deviation.

The difference between the integral deviation of traffic intensity (is the range of the first N cumulative deviations from the mean) is determined:

(6) $$R_{N}=\underset{1\leq j\leq N}{\max D_{j}}-\underset{1\leq j\leq N}{\min D_{j}}.$$

From the ratio:

(7) $$\frac{R_{N}}{S_{N}}\approx {\left(\frac{N}{2}\right)}^{H}.$$

The Hurst parameter H for network traffic is determined:

(8) $$H=\frac{\log\left(\frac{R_{N}}{S_{N}}\right)}{\log\left(\frac{N}{2}\right)}.$$

**Figure 2:**
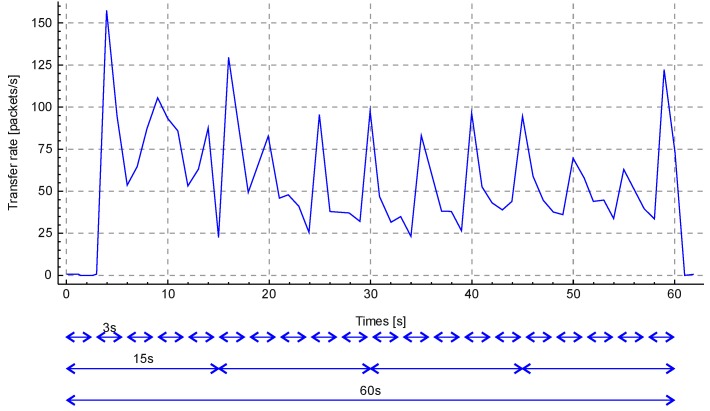
Monitoring windows (3, 15, and 60 seconds).

It is suggested to take a quantitative value of data of a certain type for 1 second per monitoring unit. Knowing the duration of the training stage (1 minute), we can propose the following scheme for calculating values: once in 5 seconds, once in 20 seconds, and once in 60 seconds. Accordingly, parameter *N* of the Formula 7 will be for 5, 2, and 60 measurements. Figure 2 shows a time span of 60 seconds with the following indications of the monitoring windows.

An important part of the method is the concept of storing reference values in tables. Tables are supposed to store data obtained after applying a mathematical formula. Each subscriber has its own table. Tables are considered expired when the device does not receive data from a subscriber within a specified timeout. A subscriber’s table stores his IP address, time when the table was created (zero point, time when the subscriber appeared in the field of view of the system), and other values that are invariable during the system operation time. In addition, the subscriber’s table stores data from three different monitoring windows (20 rows of a 3-second window, four rows of a 15-second window, and one row of a minute window). Table 1 shows an example of a subscriber’s table completed as a result of the training stage operation.

As you can see from the subscriber table example, at the end of each block, the average value for each supervised object is written. If the monitoring window is equal to 60 seconds, these values are not counted (not necessary). The standard deviation is also counted and recorded in the table. The standard deviation is counted for monitoring windows of 3 and 15 seconds.

The point of using three different types of monitoring windows is to have more accurate data for the algorithm during the detection phase.

**Figure 3:**
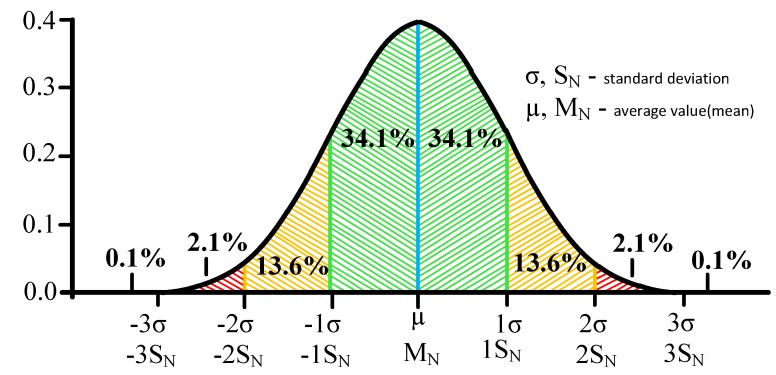
Graph of normal distribution probability density and percentage of random value hit on the sections equal to the standard deviation.

It is suggested to use the "Three Sigma" rule for determining the interval of traffic values distribution. The Three Sigma Rule states that *(3σ*) practically all values of a normally distributed random value lie in the interval (*M_N_* -*3 σ; M_N_* +*3 σ)*. More strictly, with approximately 0.9973 probability, the value of the normally distributed random value lies within the specified interval (provided that the value is true and is not obtained as a result of sample processing). If the true value is unknown, then it is not *S_N_* that should be used. Thus, the Three Sigma rule is transformed into the Three *S_N_*. Normal distribution probability density graph and random hit percentage values per segments equal to the standard deviation are shown in Figure 3.

In this case, it seems logical to suggest using remoteness from the range *(-1 S_N_;+1 S_N_*) as the traffic anomaly criterion. Thus, traffic values in the ranges of *(-∞;-1S_N_)* and *(1S_N_;+∞)* can be considered anomalous. It has been established by experience that the generated anomalies are characterized not only by a sharp increase in the values calculated by Formula 7, but also by their desire to decrease under certain conditions. In other words, the farther from (in any direction) the value obtained at the detection stage, the more likely it is that the traffic can be considered anomalous.

It is assumed that the data is sent from the analyzer to the regulator (which means that the system works with subscriber traffic already at the detection stage). The analyzer transmits the calculated value to the regulator within the minimum interval (3 seconds). The analyzer compares the calculated values with the table ones in order to fulfill the following condition:

(9) $$-1S_{N}^{}\leq H\leq +1S_{N}^{}.$$

where the *H* is the value of Hurst parameter obtained after Formula 7.

**Figure 4:**
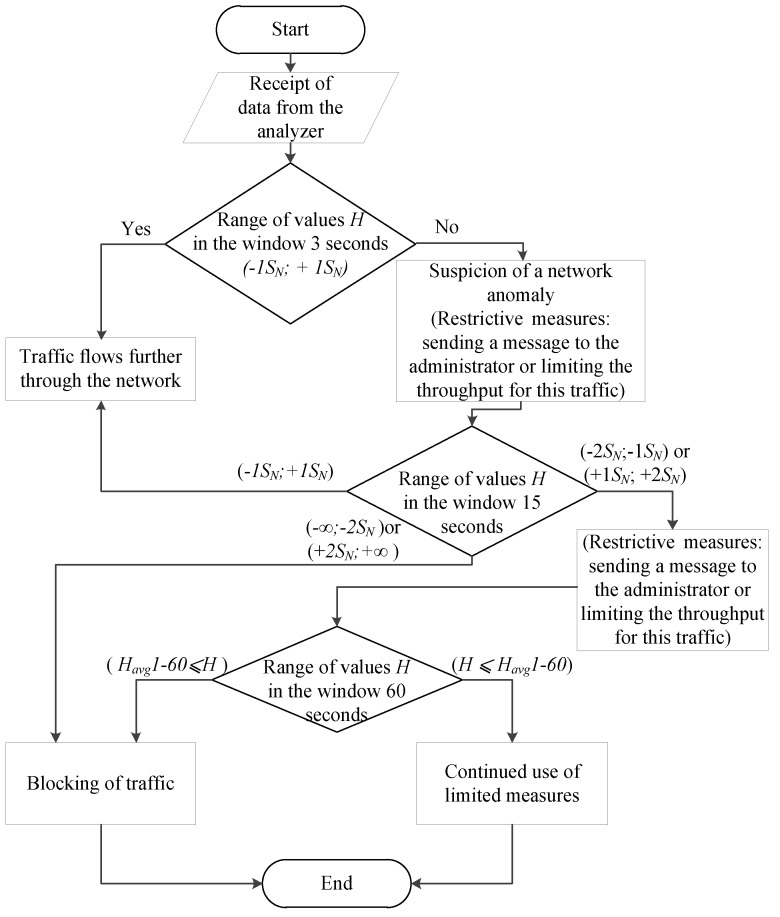
Block diagram of the detection algorithm in terms of the "Regulator" component operation.

If the condition is satisfied in the window for 3 seconds, then everything is considered to be in order. If the condition is not satisfied in the 3-second window (*H* values are outside the normal range), the system has reason to believe that there is an anomaly. However, the system will wait for the value of the 15-second window and compare it with the tables calculated in the training phase. If there is no anomaly on the 15-second window, this is considered the norm, and no action is taken. If there is an anomaly here too, it is necessary to find out how much the value deviates from the conditional norm. If the deviation range lies in *(+1 S_N_;+2 S_N_)* or *(-2 S_N_;-1 S_N_)*, restrictive measures will be taken, and the system will continue to wait for the minute window values. If the range is *(+2 S_N_;+∞)* or *(-∞;-2 S_N_)*, the system will take immediate restrictive action. In the minute window, it is possible to get only one value, so in this window, a simple comparison of *H* values of the *H* *⩽ Havg1-60* will be used as a criterion. If the declared condition is satisfied, the traffic is considered normal, if it is not satisfied, the traffic is anomalous. Below, Figure 4 shows a detailed block diagram of the algorithm operation at the detection stage. 

As you can see from the algorithm, when detecting an anomaly on the smallest monitoring window (3 seconds), the system will mark this value as suspicious, but it will not take action (since it is impossible to say with full confidence that the traffic is anomalous, given the " burst " nature of network traffic), because perhaps it is only a "dotted" anomaly. If the anomalous behavior is stored in the monitoring window for 15 seconds, appropriate measures (restrictive or prohibitive) will be taken. If the anomalous behavior is also stored on the minute monitoring window, then prohibitive measures will be taken already (Blocking of traffic). It should be noted that the application of measures is only possible in the "burst mode". Otherwise, the system passively informs the administrator about the problem (message in syslog, e-mail, etc., or limiting the throughput of this traffic to ensure QoS for real-time services).

### 3.2. Demonstration of the Anomaly Criterion H Calculation on the Example of Web Traffic

**Figure 5:**
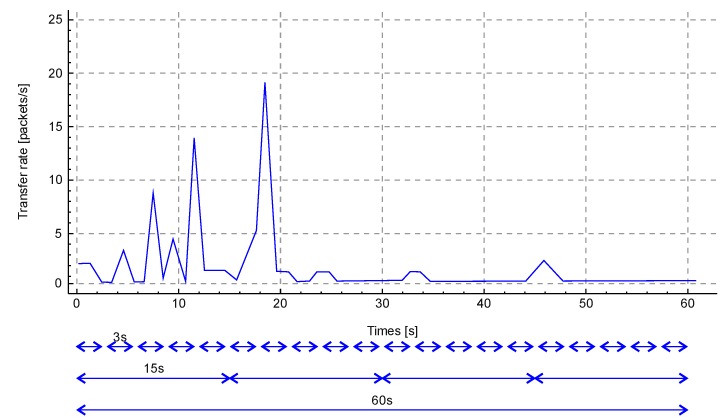
The graph of TCP (Transmission Control Protocol) packets of web traffic distribution for the monitoring period of 60 seconds.

To demonstrate the calculations, we illustrate them by the example of three-second windows for the monitoring object TCP (Transmission Control Protocol ) packets. Traffic for the demonstration of calculations is collected on the test computer in the standard mode of user work with websites. Traffic was collected using the most popular traffic analyzer Wireshark [65]. Figure 5 shows the graph of TCP packets distribution for the monitoring period of 60 seconds.

As the first input data, we get three values (2, 2, 0) in a three-second monitoring window. Using Formula (4), we get the deviation equal to 1.15. Swing is the maximum value for the monitoring window minus the minimum value. By substituting the values in Formula (7), we obtain the following

(10) $$\frac{2}{2.15}\approx {\left(\frac{3}{2}\right)}^{H},$$

(11) $$H=\log_{1.5}1.73.$$

The resulting *H* value is 1.35. This value will be recorded in the subscriber’s table. Having made similar operations for all monitoring windows, we will receive following values for TCP packets. As it is visible from Table 2, if the input data for a window of supervision are "zeros", then a calculation is not made, and dashes are input into the table. After that, the average value for each group of window values and the standard deviation are calculated.

In this example, values of the *H* parameter for web traffic are calculated in the same way the values are calculated for the other supervised objects. In the next section of this work, a number of practical monitorings will be made on the test bed using the DPI system and real traffic with examples of network attacks to test the algorithm’s performance. As a result, tables will be filled in, charts will be plotted, and conclusions will be made.

The method proposed in this section can be referred to the class of uncontrolled detection techniques. Such techniques are characterized by the fact that at the first stages (training), they do not know anything about what traffic can be considered normal and what is anomalous. Having a number of disadvantages, they still have a serious advantage over those under control, namely the potential to detect anomalies that were previously unknown.

The authors of the work suggests using the calculated Hurst parameter as the values entered in the subscriber’s table as an anomaly criterion.

## 4. Development of Software DPI System for Network Traffic Analysis and Anomaly Detection

This section discusses existing software and Open Source tools that can be used to implement the core of a software DPI system that can track channel status based on incoming traffic type data and regulate information flows. The WinDivert library is best suited for developing software for the network traffic control in the DPI system [66], because this library requires additional buffers. WinDivert implements the operation of a system driver that is installed at the kernel level between the network card and the operating system and reads packages from the kernel level buffer. Then, on the basis of the rules set by the developer, it is decided whether to send or drop the packets. 

The WinPcap and WinSock 2 library for implementing the program controller requires the use of user-level dataset cyclic buffers [67]. As a result, the creation of TUN/TAP (Tunnel/ Test Access Point virtual network adapters slow down the program, and since the DPI system must function at least with the rate of the input flow, then the rate plays an important role. However, for software development, using WinDivert, you need to undergo a certification procedure and a digital signature for drivers [68,69,70]. Certification is performed to minimize the risk of operating system crash, since WinDivert works at a kernel level where any developer error can lead to an OS (Operating System) failure. Therefore, for the development of the simulation model within the framework of this paper, the WinSock 2 library was selected. For the development of software using this library, the procedure for the digital signature of drivers is not required. In addition, the advantage of this library is that it is part of the standard Visual Studio libraries. On the basis of this library, the program traffic controller and packet capture module from the network interface were implemented for the software DPI system.

**Figure 6:**
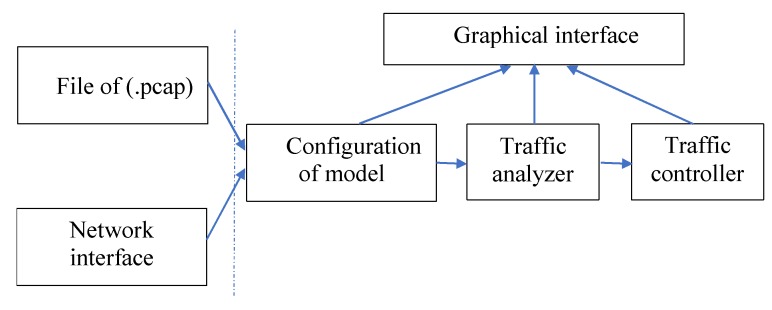
The structural scheme of the developed Deep Packet Inspection (DPI) system.

A new software DPI System for the research of effective means of controlling flows of information protocols and anomaly detection using Hurst parameter criterion has been developed based on the above considerations. The core of the system is implemented using the Microsoft Visual Studio 2013 development environment. The graphical interface is designed using the Qt 5.6.2 framework. Functions for capturing packets from network interfaces and traffic control are implemented using the WinSock 2.2 library. To write a work program, the programming language C ++ was used [71]. The structural scheme of the developed DPI system is depicted in Figure 6.

In this next subsection for DPI system implementation, algorithms for analyzing and captured traffic with protocol detection and determining statistical load parameters have been developed. In addition, algorithms that are responsible for flow regulation based on the conducted static analysis of flows and the proposed method of detection of anomalies using the Hurst parameter have been developed. These algorithms have been implemented as programs for a new software DPI system.

### 4.1. Algorithms for Network Traffic Capturing, Analyzing, and Detecting

The current trend is to move away from proprietary and closed standards to cover IP (Internet Protocol)-based sensor networks. This allows connection between WSN and the Internet, allowing smart objects to participate in the Internet of Things (IoT) [72,73]. However, building an all-IP (Internet Protocol) infrastructure from scratch will be difficult, as many different sensor and actuator technologies (both wired and wireless) have been deployed over the years [74]. The IoT sensor data are generated from various heterogeneous devices, communication protocols, and data formats that are enormous in nature [75]. This requires the development of effective tools for protocol monitoring and traffic analysis.

The traffic analyzer program is a complex system characterized by a modular construction principle and consists of the following parts: the module for reading data from the input interface; a module for parsing network and transport layer headers; protocol detectors; session tables for the protocol detector; the statistics collection module; and the graphical user interface. The input interface to the program can be any interface from which packets are received. The most common interfaces are the file and the network card interface.

The purpose of the network and transport layer header parsing module is to analyze the headers of the corresponding levels and divide the group flow into smaller flows belonging to one subscriber. If there are complex encapsulations, tunneling, and compression of the OSI (Open Systems Interconnection ) network and transport layer headers, it is necessary to first extract the primary data that were sent from one subscriber to another. Subsequently, the flow from one subscriber is divided into elementary flows and flows that are analyzed (for example, one TCP session, or several related UDP (User Datagram Protocol) sessions).

When elementary flows are selected, it is necessary to perform protocol detection and protocol type definition. DPI systems must perform this task with a very high degree of accuracy, as a detection error can lead to the user being improperly served or not served at all.

The session table for the protocol detector stores the detection result. Since a TCP or UDP session initiated by a particular application is installed and continues to exist on a particular socket, it is advisable to perform protocol detection once and enter the result into the session table, and when each subsequent packet arrives, only to look for the record in the session table.

It is reasonable to implement the session table as a tree or a hash table in order to minimize the time of searching for the required record. Thus, in the table, the information on what protocol the given package belongs to, and also if necessary certain statistical information that is necessary during the session should be stored. The key in the table is the structure containing the following fields:

- IP address of source and destination.

- Source and destination port

- Protocol (TCP or UDP)

**Figure 7:**
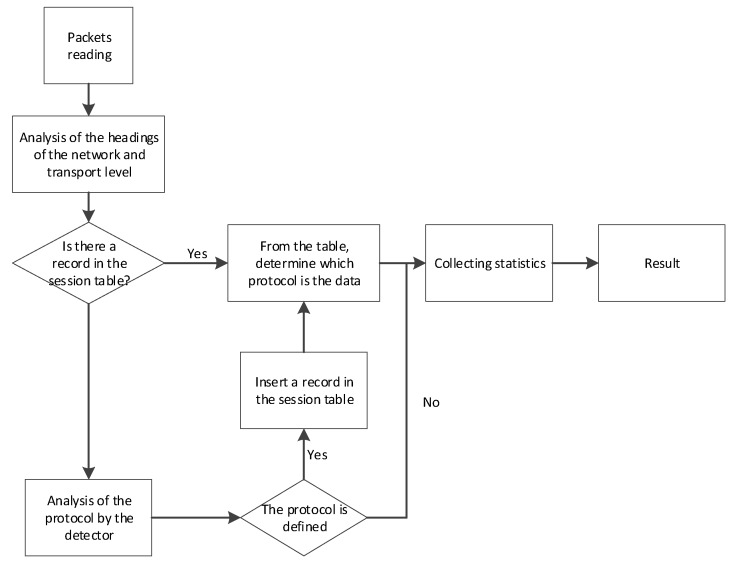
Algorithm of software system for network traffic capturing, analyzing, and detecting.

The purpose of the statistics collection module is to determine the traffic parameters, both general and for each specific protocol, including the registration of unknown traffic or traffic analyzed with errors. A general algorithm of the analyzer’s program operation is depicted in Figure 7.

Packets from the input interface are sent to the network and transport layer header analysis module, which generates a key for the session table. Then, the key is searched for in the session table, and if no record is found, the protocol is detected by the detector. All information about the packet is collected as a result of processing by each module and then is analyzed by the statistics collection module. The resulting information is presented to the user in the form of tables and graphs.

#### 4.1.1. Algorithms for Capturing Data from the Input Interface

The network traffic analyzer’s input interfaces are the computer’s network card and pcap file. All packets coming to the network card from the computer and from outside the network are captured and written to a pcap file, with a 16-byte header written before each packet, which stores the packet length and captures the packet. You can capture traffic from one or more interfaces, and you can also specify the division into files by size and time. When the network card is used as an input interface, packets are read in real time using the WinPcap library. In this case, you only need to connect the library correctly and call the interface functions.

**Figure 8:**
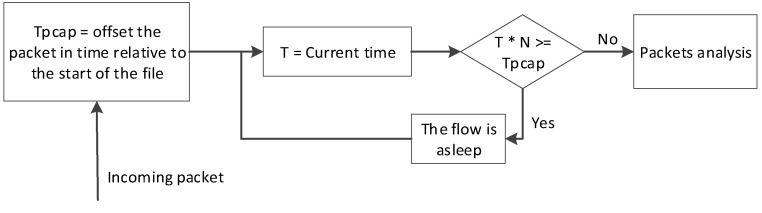
Algorithm for network packet capturing.

Capturing from a file is more complicated than capturing from the network interface. Packets are written into a pcap file at the moment when they arrive, while capturing from a file is performed at an approximately constant rate. Normal capturing from a file distorts the statistical results because it does not take into account packet writing to the file. For the results not to differ when reading from two interfaces, you need an additional algorithm so that the moments of the beginning of analysis of each next packet are fixed, as shown in Figure 8.

According to the algorithm, the worker flow starts to process the packet if the program’s running time at the current moment is N times less than the time stamp of the packet in the file. Otherwise, the thread will wait and check the condition again.

#### 4.1.2. Development of Protocols Detector

Without the protocol detector, the DPI system is unable to do almost anything, because the function of the detector is to distinguish between protocols and applications for further statistics collection. Historically, there are thousands of protocols on the Internet, for each of which you need to develop a separate detection algorithm. Most of the protocols are not standardized, and the development of protocols in the best case is in accordance with the documents RFC (Request for comments), which complicates the development of protocol detectors, because the RFC in many cases is only a recommendation. An organization developing a particular application does not necessarily have to fully comply with the RFC, but it may have to make some adjustments.

Detecting protocols is complicated by the fact that most companies developing protocols for their private purposes do not disclose details of protocol implementation, and in free access, at best, there are only presentations that describe all the benefits of using this protocol. Very often, the document description of the protocol is available only for employees of the company or developer.

If the protocol is not described, the developer should investigate the principles of its work himself, because it is only possible to write a detector that will have high performance and low probability of error when the developer knows the process of interaction between network devices and the structure and parameters of the packages.

The set of fields forms the uniqueness of a particular protocol and, due to this, you can write a protocol detector. However, in conditions of high-speed traffic with algorithms of protocol detection, there are strict requirements for performance, because the program must process large amounts of data and create a minimum transmission delay. Detection algorithms must be both simple and robust, so that the probability of a false alarm is minimal [76].

**Figure 9:**
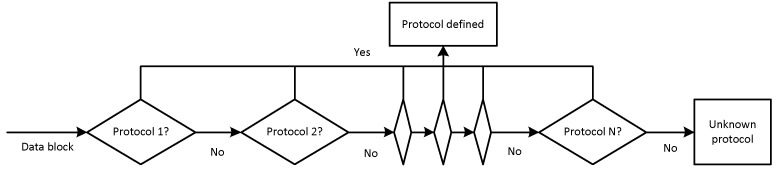
Sequential algorithm of detection.

**Figure 10:**
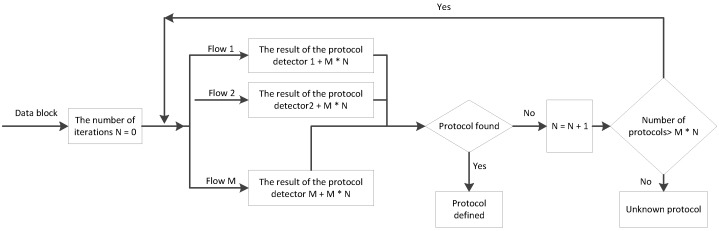
Parallel detection algorithm.

The detection algorithm can be of two types:

- Sequential (Figure 9)—used for low-speed data flows. Each of the detection algorithms is executed when the execution of the previous algorithm is blocked. One processor thread is used.
- Parallel (Figure 10)—for high-speed data flows. Detection algorithms are executed in parallel, each in its own processor thread. When each thread has finished executing its own algorithm, if the protocol is defined by one of the algorithms, the execution stops; if not, each thread is provided with a new algorithm for execution.

Algorithm for DNS protocol detection

**Figure 11:**
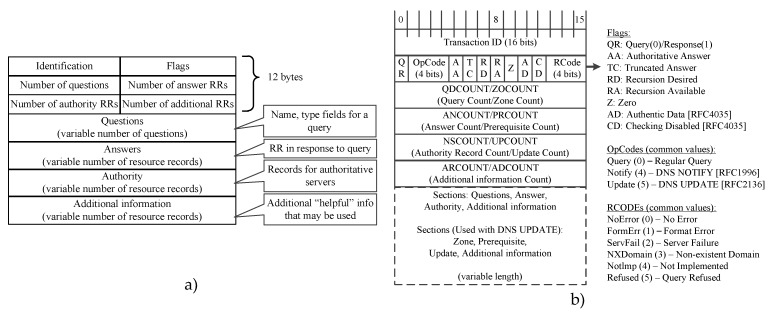
DNS message format (**a**) and the flag field in the DNS header (**b**).

DNS (Domain Name System) is an application layer service protocol, without which other protocols cannot work. So, DNS packets are always present among network traffic. Therefore, it is worth adding the algorithm of DNS protocol processing to the traffic analysis system. DNS detection consists in comparing the structure of the incoming packet and the structure of the DNS packet. The format of the DNS message is depicted in the Figure 11a [77]. The message contains a 12-byte header followed by four variable length fields [78].

The value in the identification field is set by the client and returned by the server and allows determining which request has received a response. The 16-bit field of the flags is divided into several parts, as shown in Figure 11b.

The flag field consists of the following main fields:

- QR (Query Response)(message type) field with the size of 1 bit 0 denotes the request, 1 denotes the response
- The zero field is 3 bits, which should be equal to 0.
- Rcode is a 4-bit return code field. It takes the following values: 0 (no error) and 3 (name error).

The following four 16-bit fields indicate the number of entries in four variable length fields. In a query packet, the number of questions is usually 1, and the counter for the other fields is 0. In a query packet, the number of answers is at least 1, and the other two counters can be either zero or non-zero.

**Figure 12:**
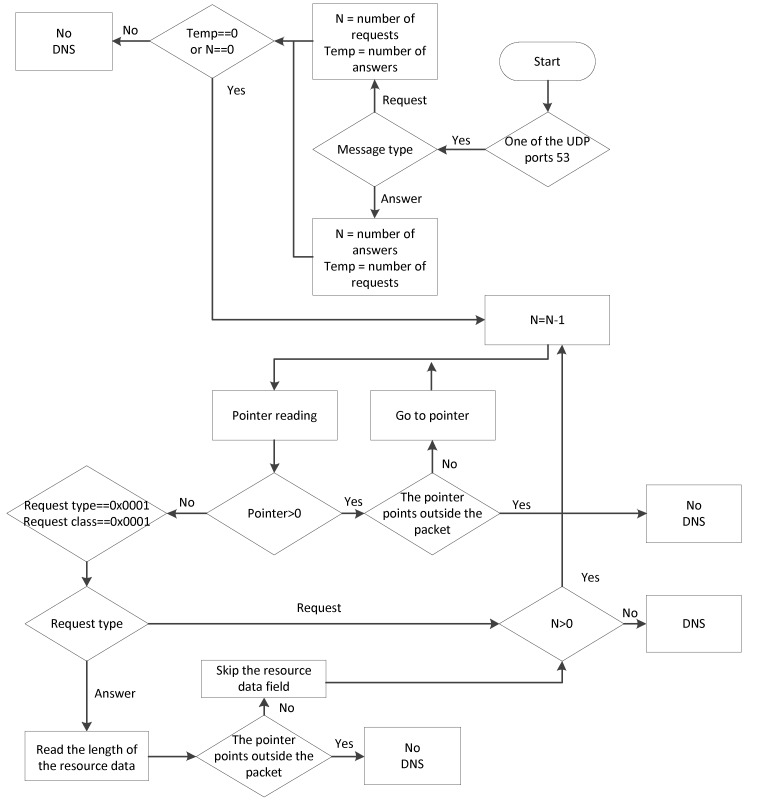
The algorithm of the DNS protocol detector.

So, knowing the structure of the DNS packet fields, you can make a detector that will discard packets with impossible field values. If the packet passes all the checks, it is DNS. The algorithm of the DNS protocol detector is shown in Figure 12. 

Despite the complexity of the block diagram, the algorithm must work quickly, because no complex operations are performed, except to check the content of fields. The sequence of the algorithm’s actions is as follows:

1) Checking the port. UDP and TCP ports number 53 are reserved for DNS protocol. Although in most cases, DNS packets are transferred through UDP, and support for TCP operation is one of the main requirements for the DNS server.

2) Check the number of requests and number of responses fields. If the type of message is a query, then the response field will be empty; if the type is a response, then the query field will be empty.

3) Make as many iterations as the number of queries/responses, reducing the number of queries/responses by 1 at each iteration. When the value 0 is reached, we exit the loop and make a decision. At each iteration, we parse the domain name—that is, we read the pointers and go to the values they specify. Exiting the package means that it is not DNS. Zero pointer means the end of the domain name. The request type and class for such packets should be 0 x 0001.

4) If the packet type is the answer, you should read the length of the resource data and navigate by the pointer.

5) When the query/answer variable reaches the value of 0, it is decided that this is a DNS packet.

Algorithm for RTP protocol detection

The RTP (Real-time Transport Protocol)protocol transfers in its header the data needed to collect audio or video from the receiving node, as well as data on the type of information encoding (JPEG, MPEG, etc.). The header of this protocol transmits the timestamp and package number. These parameters allow you to determine with minimal delay the order and moment of decoding of each packet and interpolate lost packets.

RTP has no standard reserved port number. The connection is basically established on an even port, and the next odd number is used for communication via RTCP (Real-Time Transport Control Protocol), which is used for feedback from recipients. An RTP session is established for each media stream. The session consists of an IP address and a port pair for RTP and RTCP. Audio and video streams will be transmitted through different RTP sessions. The ports that form the session are connected to each other by means of other protocols, such as SIP(Session Initiation Protocol) and RTSP (Real Time Streaming Protocol). The RTP protocol has a variable length header (Figure 3.13). The minimum length of the header is 12 bytes and consist of fields [79]:

V (2 bits). Version field. The current version is the second one.

P (1 bit). Field of filling. This field indicates that octets are being filled at the end of the payload. Filling is used when the application requires that the payload size be a multiple of, for example, 32 bits. In this case, the last octet indicates the number of filling octets.

X (1 bit). Header extension field. When a bit is set, the main header contains another additional one that is used in experimental RTP extensions.

CC (4 bits). Sender number field. This field contains the number of sender identifiers, whose data is in the package, and the identifiers themselves follow the main header.

M (1 bit). Marker field. The purpose of this field depends on the payload. A token bit is usually used to indicate the boundaries of the data stream. In the case of video, it denotes the end of the frame. In the case of voice, it specifies the beginning of speech after the silence period.

PT (7 bits). Field of payload type. This field identifies the payload type and data format. In a stationary state, the sender only uses one payload type during a session, but can change it in response to changing conditions if RTCP signals this.

Sequence Number (16 bits). Sequence number field. Each source begins to number packets with an arbitrary number, increasing the sequence number by one with each RTP packet sent. This allows the loss of the packets to be detected and the packets to be re-sorted if there is a mix. Multiple consecutive packets can have the same timestamp if they are logically generated at the same time, such as packets belonging to the same video frame.

Timestamp (32 bits). This is the timestamp. This field contains the value of the moment of time (mostly in counts as a sample rate) at which the first payload data octet was created.

Synchronization Source (SSRC) Identifier (32 bits) and Synchronization Source Identifier Field. A random number that identifies the source of a session and is independent of the network address. This number plays an important role in processing data from a single source.

**Figure 13:**
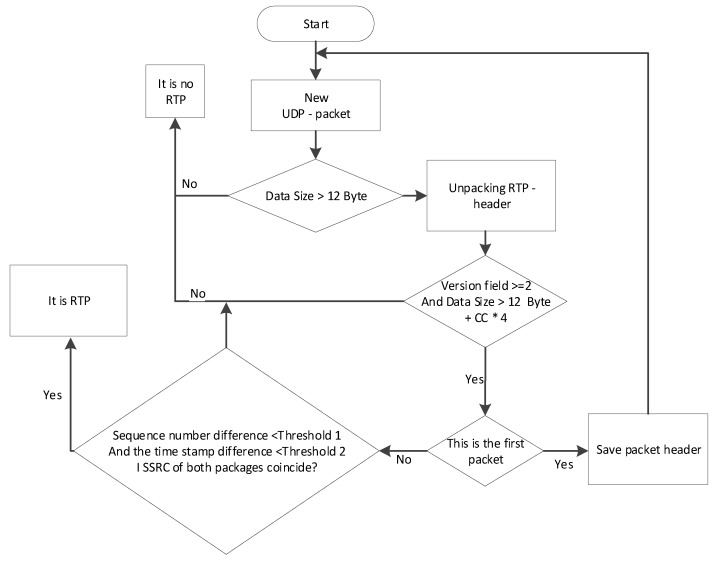
The algorithm of the RTP protocol detector.

Contributing source (CSRC) Identifier (32 bits). A list of source identifier fields that are "mixed" into the main stream, for example, using a mixer. The mixer inserts a whole list of SSRC source IDs that were used to build this RTP packet. This list is called the CSRC. The number of items in the list is from 0 to 15. If the number of participants is more than 15, the first 15 are selected. An example of this is an audio conference where the RTP packet contains the voice data of the participants, each of which has its own SSRC. Since the RTP header has no clearly defined fields except for the version field, it is impossible to use classical pattern analysis. However, the problem of detection of RTP protocol is easily solved with use of the behavioral analysis of the protocol, despite the value of separate fields of the protocol. However, the behavioral analysis, unlike the signature analysis, always requires more memory, because there is a need to save the package header. The algorithm of the RTP protocol detector is depicted in Figure 13.

For each UDP packet, two simple checks must be performed: the version must be at least 2 and the length must be sufficient to parse the header. This check will filter more than half of the UDP packets. The packet header and packet information that has passed the simple check are stored in the detector. When two packets (or more if necessary) are received, three fields are analyzed.

The difference in the ordinal numbers of the packets is small. The threshold value should be selected depending on the probability of losing packets. The higher the probability of loss, the higher the threshold should be selected, but the higher the probability of error.

The difference in time stamps (samples) should be less than a certain threshold value. It depends on the type of traffic (audio/video) and the difference in order numbers. Therefore, if you know the flow parameters, you can estimate the maximum possible value of this field. If the flow parameters are unknown, the default values should be used. The SSRC of all packets must be the same.

Algorithm for HTTP protocol detection

HTTP (Hyper Text Transfer Protocol) is an application layer data transfer protocol and is the primary protocol for obtaining information from websites. The HTTP protocol uses client–server technology: the client sends a request and initiates the connection; the server that receives the request, processes the request, and sends the result to the client. An example HTTP header is shown in the work [80,81]. 

Each HTTP message consists of three parts, which are transmitted in the specified order: the start line defines the type of message; headers are useful information that characterize the message body, transmission parameters, and other information.)

The message body is in HTML (HyperText Markup Language )code for displaying it in a browser. Each of the headers must be separated by an empty line. HTTP is an unsecured protocol. Anyone can access information by intercepting it. For example, an operator can intercept information from a client connected to it. HTTPS is an extension of the HTTP protocol and is an HTTP protocol with TLS(Transport Layer Security) encryption. TLS protocol is used to create eavesdropping-proof and information-substitution connections between network nodes. It is suitable for transmitting any amount of data in both directions, as well as checking that the data exchange takes place exactly between these nodes, for which the channel was planned. These tasks are called, respectively, ensuring the confidentiality, integrity, and authenticity of the connection. TLS, as well as usual HTTP, works on TCP protocol. By the beginning of data transfer, the client and the server should carry out the initialization process.

As TLS works on TCP, between a client and a server, TCP connections (SYN–SYN; ACK–ACK) are established. The client sends a specification to the server specifying the version of the protocol it wants to use, supported encryption methods, etc. (ClientHello). The server confirms the version of the protocol used, selects the encryption method from the list provided, attaches its certificate, and sends the response to the client in the form of several commands (ServerHello—Certificate—ServerHelloDone). The client checks the received certificate and initiates the key exchange using the RSA (Rivest-Shamir-Adleman) algorithm or the Diffi–Hellman protocol, depending on the set parameters. It then sends a final message (ClientKeyExchange—ChangeCipherSpec—Finished). The server processes the message received from the client, checks the MAC (Media Access Control Addres), and sends the final (Finished) message to the client in encrypted form. This is followed by the transfer of encrypted data (Application Data). The data is transmitted to TLS in the form of TLS records, each of which consists of a header containing such fields: (data type (1 byte); Version (2 bytes); TLS now has such versions: 0x0300, 0x0301, 0x0302, 0x0303; Data length (2 bytes); Data field). After the record’s data field, there are blocks, each of which has a structure that depends on the record type. For example, in record with 0x16 type (TLS handshake), which is contained in the first package of session, blocks will have the following structure: (Type (1 byte)—message from client (code 0x01) or from server (0x02); Length (3 bytes); Version (2 bytes)).

**Figure 14:**
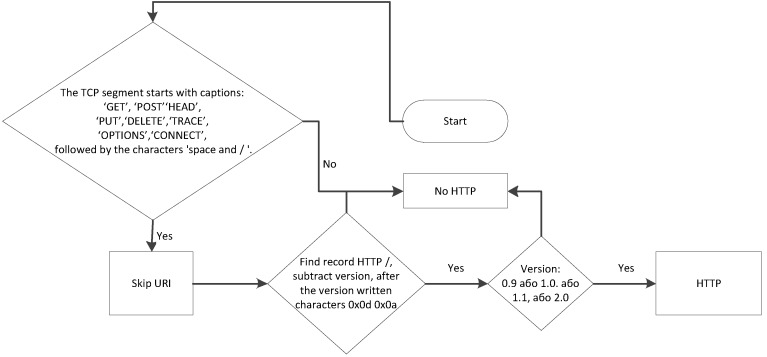
The algorithm of the HTTP protocol detector.

Since the HTTP protocol has a clearly defined structure, it is not difficult to detect. The HTTP session starts with the client sending to the server one of the known requests, i.e., TCP; the segment must start with one of the commands, after which the URI (Uniform Resource Identifier) address and version are written. The algorithm of the RTP protocol detector is depicted in Figure 14.

Algorithm for TLS protocol detection

**Figure 15:**
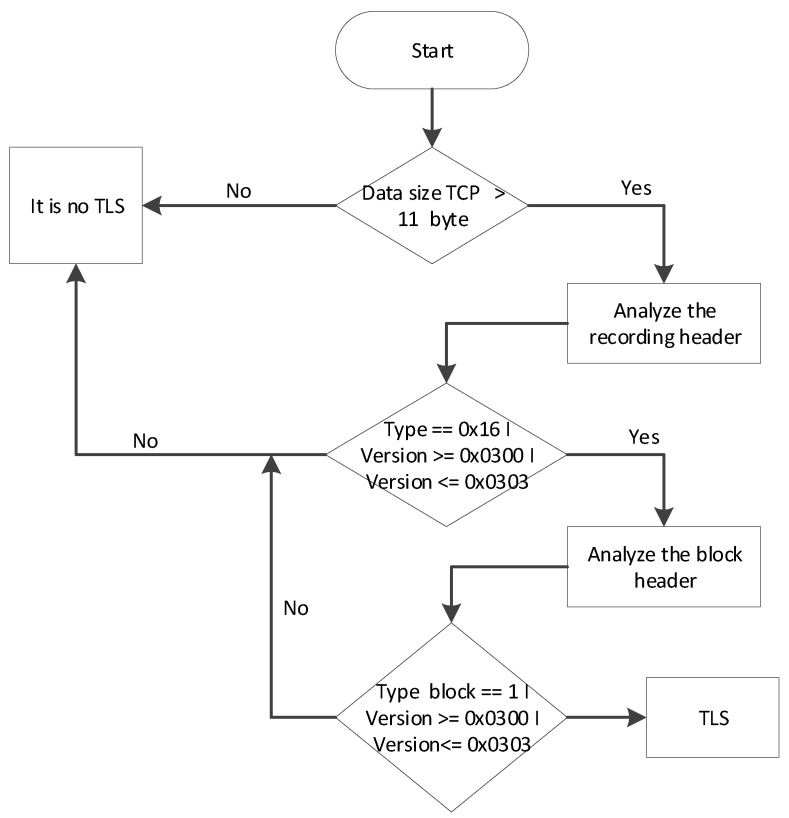
Algorithm for TLS (Transport Layer Security) protocol detection.

The detection of TLS protocol is carried out by the first packet, which is sent from the client to the server. This package contains a 5-byte record header followed by a 6-byte block header (Figure 15). So, the TCP size of a package should be not less than 11 bytes, the versions specified in the headings should correspond to the actual version of the protocol, and the record type and block accordingly should be equal to 0 × 16 (TLS handshake) and 0 × 01 (TLS client handshake). The algorithm for TLS protocol detection is depicted in Figure 15.

Algorithms for BitTorrent and uTorrent Transport Protocol (uTP) detection

BitTorrent and uTorrent Transport Protocol (uTP) are now the primary protocols for file transfer in peer-to-peer networks [82,83]. When files are transferred, they are broken up into small pieces and transmitted as such. The torrent client downloads all parts and then collects the file. The main difference between the torrent protocols and FTP (File Transfer Protocol) is that during the slice download process, the client immediately gives access to the slices for downloading to other members of the network and allows the torrent files to be transferred at a much faster speed. 

The disadvantage of the BitTorrent protocol is that it works over TCP and therefore torrent traffic will slow down other applications, such as the browser, mail client, etc., which are much more important for the user.

The uTP protocol is an alternative to the BitTorrent protocol. It works over UDP, which will result in torrent traffic being transmitted with a lower priority than TCP traffic. At the same time, data transmission via uTP is more effective, through a smaller volume of service traffic.

The uTP header structure consists of the following fields:

- Type of package. This field can take values 0–4;
- Protocol version. For current version of the protocol 1;
- Extensions. This field is like TCP options. If this field is not zero, the extension field will be placed after the uTP header;
- The connection identifier. This field contains a random number that all packets belonging to a particular connection have;
- Time stamp. Contains the sending of the packet in microseconds;
- The difference in the time stamps. Defines the time a packet is transmitted over the network;
- The size of the window. Defines the number of packets that can be transmitted between hosts without confirmation;
- Sequential number of packets. Determines the current number of the packet;
- The number of the confirmed packet. Determines the last packet to which the confirmation was received.

The detection of the uTP session should be performed on the first packet in this sequence: first check whether the data size is large or equal to 20 bytes. Then parse the header, and check the version and value of the type. Since the first packet in the session cannot yet have the timestamp difference field set, it should contain 0. The connection ID must not be 0, and the extension field must not contain a value greater than 8.

**Figure 16:**
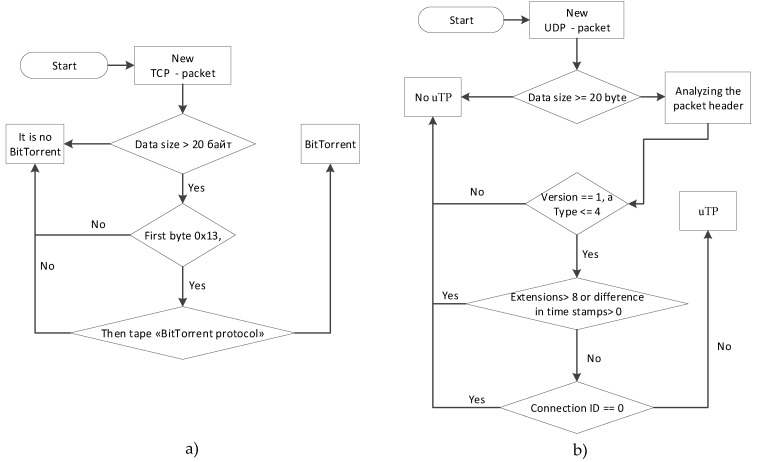
Algorithms for BitTorrent (**a**) and uTorrent Transport Protocol (uTP) (**b**) detection.

The algorithms for BitTorrent and uTP detection are depicted in Figure 16.

#### 4.1.3. Statistics Collection Algorithm

Statistics are collected using a basic data structure *(Stat)*, a copy of which is used to collect statistics from one of the protocols. The fields of the structure are as follows: total number of bytes *(N)*; number of bytes after last bandwidth recalculation *(n)*; time of last bandwidth recalculation *(T)*; and last throughput value *(Thr)*.

To collect statistics on all protocols, an array of Stat structures is used, the size of which is equal to the number of protocols that are processed plus 2, because it is necessary to keep the general statistics and statistics on unknown traffic. In addition, a separate array of Stat structures must be stored for each subscriber.

**Figure 17:**
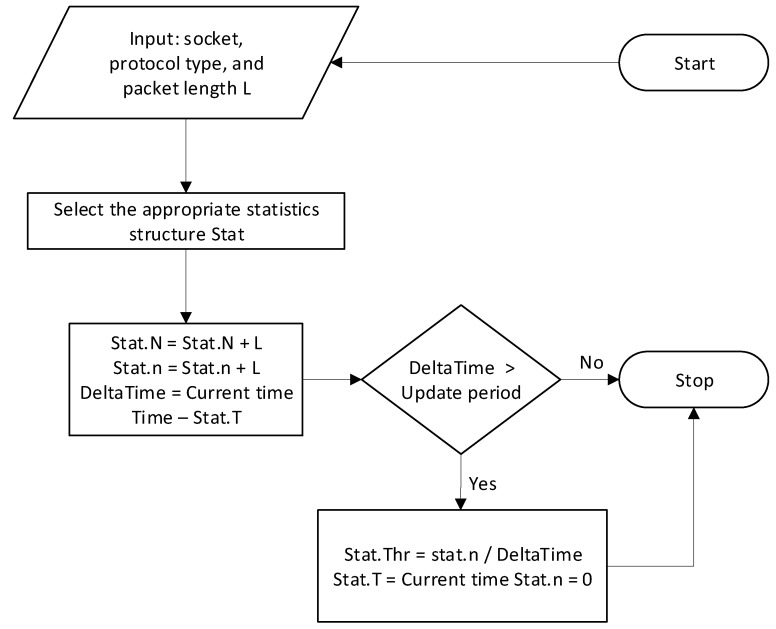
Statistics collection algorithm.

The statistics collection algorithm is depicted in Figure 17.

The input data for the statistics collection system is a socket, a protocol type, or an unknown type and socket. According to this, the Stat structure of statistics is selected, in which we update the values of fields *N*, n of the Stat structure according to the value *L*.

The throughput is calculated at the moment of time corresponding to the chart update period.

### 4.2. Algorithms for Network Traffic Capturing, Analyzing, and Detecting 

This model implements the regulation of information flow by protocols. Decisions on blocking, restriction, or priority granting are made depending on the protocol type and load is created by it. For the definition of speed of an incoming stream, the following algorithm is applied. The algorithm is executed during the whole time of the program operation and is used to measure the load of input streams. Each socket is treated as a separate data stream. For each data stream, the number of input data in bytes per time unit is recorded.

At the beginning of the algorithm, the program’s running time is recorded, and the timeline is set. For the timeline division, 1000 ms is taken. Then, the algorithm goes to step 1.

Step 1. Find the difference in time between the start of the program and real time; if this difference is greater or equal to the price of the scale, then proceed to step 2. If the difference in time is less than the price of the division, then proceed to step 3.

Step 2. The instantaneous load values are passed to another flow of the program that is responsible for controlling the flow, the global time stamp is assigned a real-time value, and the instantaneous load values are set to zero. Go to step 3.

Step 3. Check if the new packet has arrived at the interface’s input; if it does not, go back to the previous step. In the event that a new packet arrives, step 4 is executed; otherwise, go back to step 1. 

Step 4. Read the packet’s type and its length in bytes. We sum up the load for a specific type and the total load. Go to step 1.

**Figure 18:**
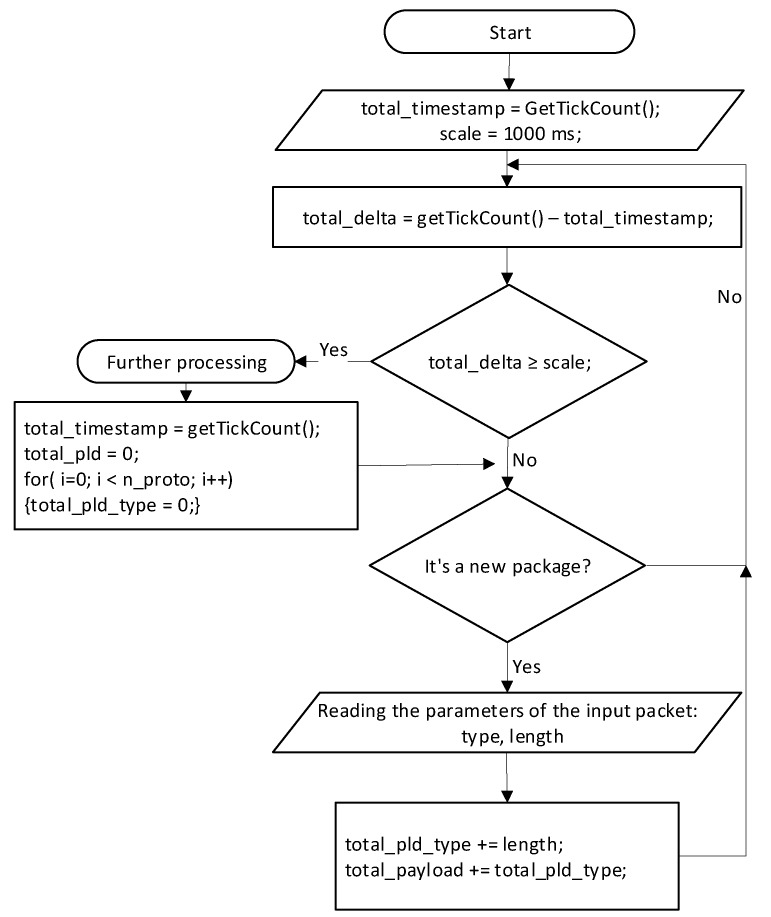
Algorithm for determining the load of information flows.

The algorithm for determining the load of information flows is depicted in Figure 18.

Determining the load of a specific type of traffic can be expressed by the following formula:

(12) $$C_{П}=\sum_{i=0}^{n}C_{{}_{{}_{{П}_{i}}}},$$

where n is the number of flows of the same type, and $C_{{}_{{}_{{П}_{i}}}}$ is the load is created by one flow of a certain type.

The total load on one interface is calculated by the formula:

(13) $$C=\sum_{i=0}^{N}C_{i},$$

where *N* is the number of types of load on one interface, and $C_{i}$ is the load is generated by one type of traffic.

This model can function in two modes:

1) Blocking. When used this mode, all traffic that was identified as "harmful" (as an example of harmful traffic, torrent traffic was selected) is blocked and does not pass through the interface. This mode should be used when "harmful" traffic occupies a large part of the overall bandwidth and because of this subscribers who use other services such as video or audio communications do not receive the appropriate quality of service.

2) Traffic Prioritization. When this mode is used, "malicious" traffic is not blocked completely. Priority is given to real-time data. That is, "harmful" traffic is blocked only when its passage leads to the loss of real-time data; otherwise, the traffic is not blocked. This mode is used when the load of malicious traffic on the interface is insignificant but can lead to deterioration of traffic service quality.

**Figure 19:**
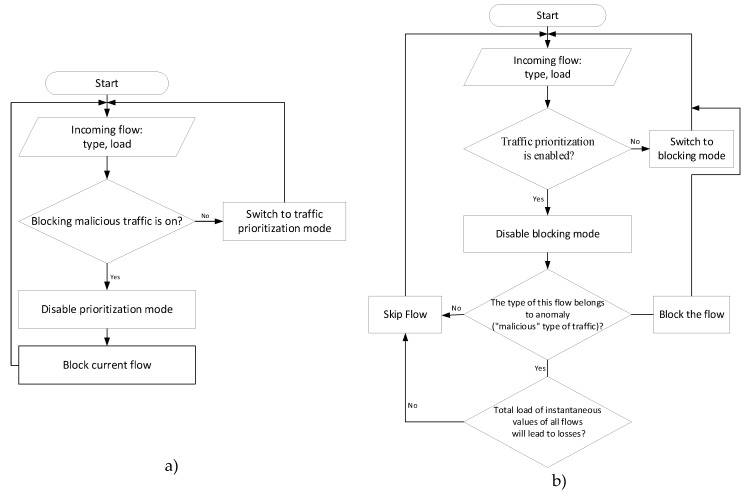
Algorithm of anomaly blocking (**a**) and traffic prioritization (**b**) mode.

An algorithm of anomaly blocking and traffic prioritization is depicted in Figure 19.

The choice of the system operation mode allows increasing the flexibility of the transmission channel and changing its behavior not only depending on its load, but also on the type of data being transmitted.

## 5. Experimental Data and Result Analysis

To evaluate the performance of the developed system, we compared the proposed DPI software system with the existing SolarWinds deep packet inspection system to detect and prevent network traffic anomalies. SolarWinds Network Performance Monitor (FREE TRIAL) is a network monitoring tool that includes deep packet inspection to identify the source and destination applications and endpoints on network traffic. The purpose of DPI in the SolarWinds tool satisfies two aims of network administrators. The first is to identify the types of traffic that use up most of the system’s resources. Excessive load on the network makes the working environment difficult for everyone, and it is important to find out exactly where all of that demand originates. 

Deep packet inspection also gives the Network Performance Monitor security functions. DPI techniques will identify specific users and applications that cause surges in traffic and display erratic behavior. Those peaks in demand could be caused by hacker attacks; however, they could also be caused by business requirements, such as end-of-the-month account processing. DPI lets you see whether those surges are generated by legitimate business activities. Irregular behavior can be blocked. 

For this purpose, the authors have developed an experimental stand of a real network to test and compare the SolarWinds DPI system with the proposed DPI system using the above algorithms and anomaly detection method based on Hurst parameter estimation.

### 5.1. Test Bed for Network Traffic Analysis and Anomaly Detection

The scheme of the experimental stand consists of the following basic elements: end devices (IoT camera, laptop, and computer) that generate legitimate traffic; the generator of non-legitimate traffic (network attack), which is a conventional computer on the corporate network [84]; network devices on the corporate network (switch and router), and the object of attack, which is a standard web server on the Internet and installed before the router software DPI system.

**Figure 20:**
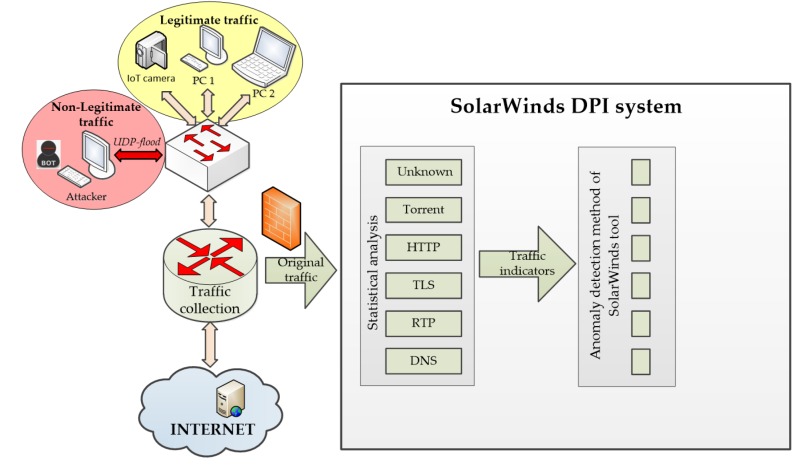
Network traffic analysis and anomaly detection scheme using the SolarWinds DPI system.

**Figure 21:**
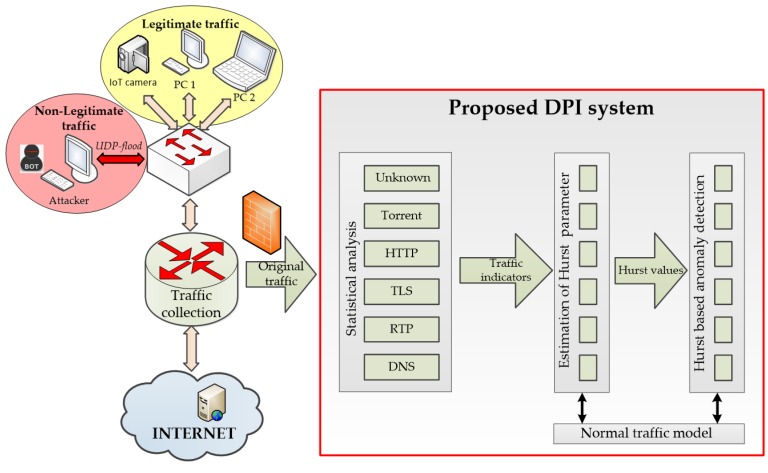
Network traffic analysis and anomaly detection scheme using the proposed DPI system.

The network traffic analysis and anomaly detection scheme using the SolarWinds DPI system is depicted in Figure 20 and the scheme using the proposed DPI system is depicted in Figure 21.

For further analysis and comparison of the systems, the file capture mode (pcap file) is used to capture the network packets from all subscribers, including non-legitimate traffic and the variety of protocols they create. By capturing the packets, it is possible to investigate the same aggregated traffic for the two systems to obtain accurate results. To demonstrate the system in the mode of capturing packets from a file, set the bandwidth of the router interface at 40 Mbit/s. Most of the legitimate traffics are created by protocols such as RTP, Torrent, HTTP, TLS, and other types. A variant of the popular UDP flood attack was chosen as the non-legal verification traffic [80]. The essence of this attack is to send multiple UDP packets (usually, large ones) to certain or random port numbers of the remote host, which for each received packet must identify the corresponding application, make sure that it is not active, and send a response ICMP (Internet Control Message Protocol) message: "the recipient is unavailable". As a result, the attacked system will become overloaded: the UDP protocol does not have an overload prevention mechanism, so after the attack starts, the parasitic traffic will quickly become overloaded. It will capture all available throughput, and only a small fraction of the traffic will remain useful. 

In our work, we have generated Non-Spoofed UDP Floods that are difficult to detect. The scheme of organizing a Non-Spoofed UDP Flood attack is the same as in the case of UDP Flood. The difference is that UDP packets are generated from real IP addresses by bots, which makes it very difficult to combat this type of attack, especially if the bots generate traffic due to NAT (Network Address Translation), behind which there are legitimate users. Just like a regular UDP Flood, this type of attack aims to exhaust system resources and fill the network channel with "malicious" traffic. This type of attack is harder to identify because it resembles good traffic [84,85].

Filtering UDP traffic during such an attack is quite a complex task, so most operators offer only one solution: blocking the victim server to save the rest of the network. In our work, we offer an approach that can detect an attack and take necessary actions to filter it without blocking the attacking server. Nothing has changed in the testing scheme. At the first stage, the user’s usual work on the computer (DNS queries, etc.) is simulated. The second stage starts the packet generator using the hping3 utility with the following parameters: hping3 -q -n -a 192.168.0.101 --udp -s 53 --keep -p 68 --flood 192.168.0.100. 

Before legitimate and non-legitimate traffic arrives at the router’s input port, it is analyzed by the DPI system, and after analysis, it is forwarded to the router’s output port. At the router output, the traffic is monitored with the help of the developed program both in the experiment with the proposed DPI and the existing SolarWinds DPI system.

#### 5.1.2. Traffic Analysis and Anomaly Detection Results Using Solarwinds DPI and Proposed DPI System (Transmitting Only Legitimate Traffic)

**Figure 22:**
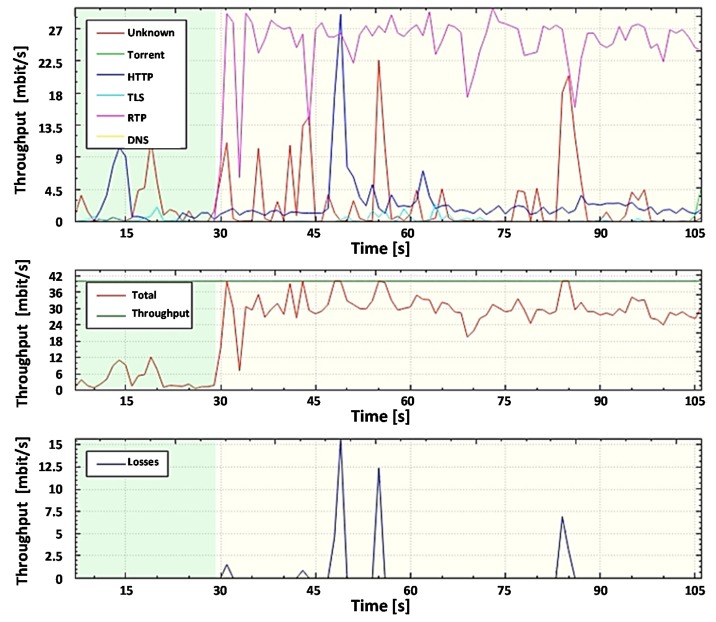
Throughput monitoring, in the transmission of legitimate traffic using the existing SolarWinds DPI and the proposed DPI system.

The first stage of the experiment was to evaluate the router’s throughput while transmitting only legitimate traffic under the existing SolarWinds DPI and proposed DPI systems usage conditions. Both systems showed the same throughput utilization results (Figure 22). 

As you can see from Figure 22, at the initial stage (salad color), the load on the interface is insignificant and fluctuates at the level of 2 Mbit/s, and the loss level of 0% of the total load. This period shows the work of the channel at low loads. In this section, most of the load is generated by HTTP, TLS, and unknown traffic. On further monitoring (shown in yellow), you can see that the load on the interface is increasing due to the growth of RTP traffic and fluctuates at the level of 38 Mbit/s with a total loss of 1.8% of the total. This section shows the channel operation under peak loads. It should be noted that the losses in this segment are caused by burst traffic and are not constant.

#### 5.1.3. Traffic Analysis and Anomaly Detection Results Using Solarwinds Dpi System (Transmitting Legitimate and Non-Legitimate Traffic)

The second step of the experiment was to evaluate the router’s throughput when transmitting legitimate and non-legitimate traffic (Non-Spoofed UDP Floods attack) using the existing SolarWinds DPI system.

**Figure 23:**
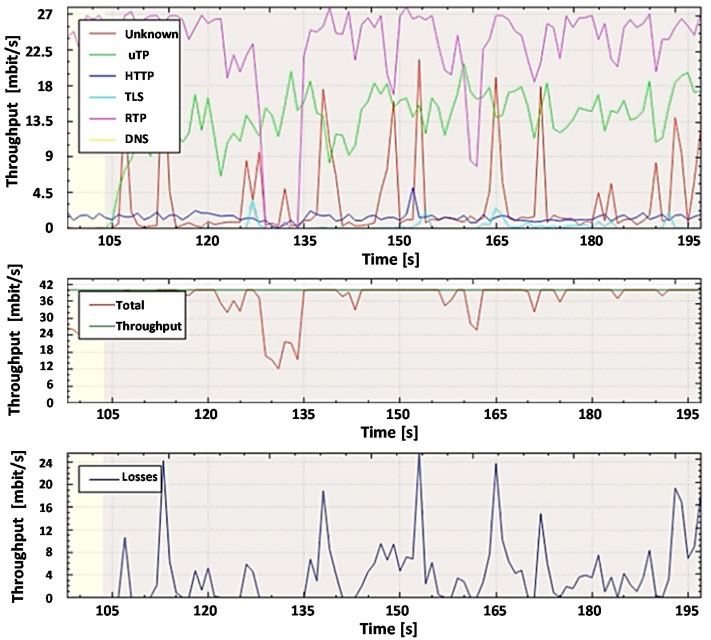
Throughput monitoring, in the transmission of legitimate and non-legitimate traffic (Non-Spoofed UDP(User Datagram Protocol) Floods attack) using the SolarWinds DPI system.

On the output interface of the router when using the SolarWinds DPI system, torrent traffic (uTP) works over UDP, which appeared in the channel (the figure is highlighted in brown). The SolarWinds DPI system detection Non-Spoofed UDP Floods attack as an legitimate uTP traffic. Throughput monitoring in the transmission of legitimate and non-legitimate traffic (Non-Spoofed UDP Floods attack) using the SolarWinds DPI system is depicted in Figure 23.

As you can see from Figure 21, this leads to significant losses of other types of traffic, such as RTP and HTTP; the level of total losses rose to 7.2%, and the maximum percentage of current losses was 48%, also, the nature of losses from jumped to constant. This indicates that up to 48% of the payload is lost every second. Such losses are unacceptable for holding, for example, video conferences, IP telephony, or comfortable surfing on the Internet.

#### 5.1.4. Traffic Analysis and Anomaly Detection Results Using Proposed DPI System (Transmitting Legitimate and Non-Legitimate Traffic)

The third step of the experiment was to evaluate the router’s throughput when transmitting legitimate and non-legitimate traffic (Non-Spoofed UDP Floods attack) using the proposed DPI system. After the procedure of traffic generation and value table construction, calculations were performed according to the formulas and ideas proposed in the work. The results of these calculations are presented below. For each group of test data, a range *(-3S_N_;+3S_N_)* was found, and the statistical significance of differences in mean values was assessed. Tables of subscribers for UDP packets for "normal" class are showed in Table 3.

It is assumed this table (as in the first experiment) will be the reference one for the UDP object. In this case, the system will compare data obtained during the detection phase with that from the table. The "anomaly" class table is given in Table 4. It is given for illustrative purposes only, and the system does not use such type of tables.

**Figure 24:**
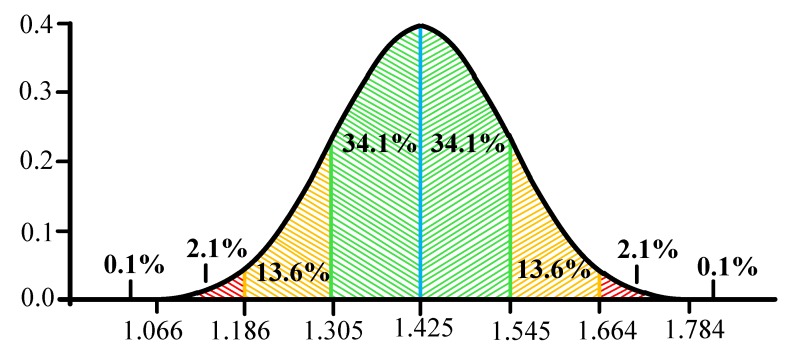
Range of values (-3S_N_;+3S_N_) for normal UDP traffic for monitoring windows = 3 seconds.

As before, the table for the "anomaly" class serves as an auxiliary material for clarity, as well as a basis for charting. Figure 24 shows the range of values *(-3S_N_;+3S_N_)* for normal UDP traffic for monitoring windows = 3 seconds.

**Figure 25:**
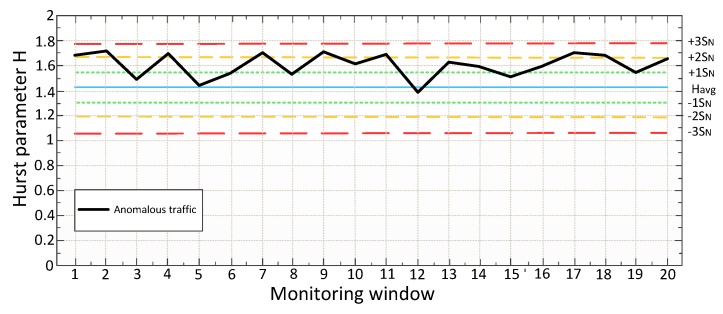
Values in 60 seconds with 3-second monitoring window for "anomaly" class (Non-Spoofed UDP Floods attack).

Figure 25 shows in which of the monitoring windows the values of anomalous traffic exceeded the threshold and in which range they fell.

**Figure 26:**
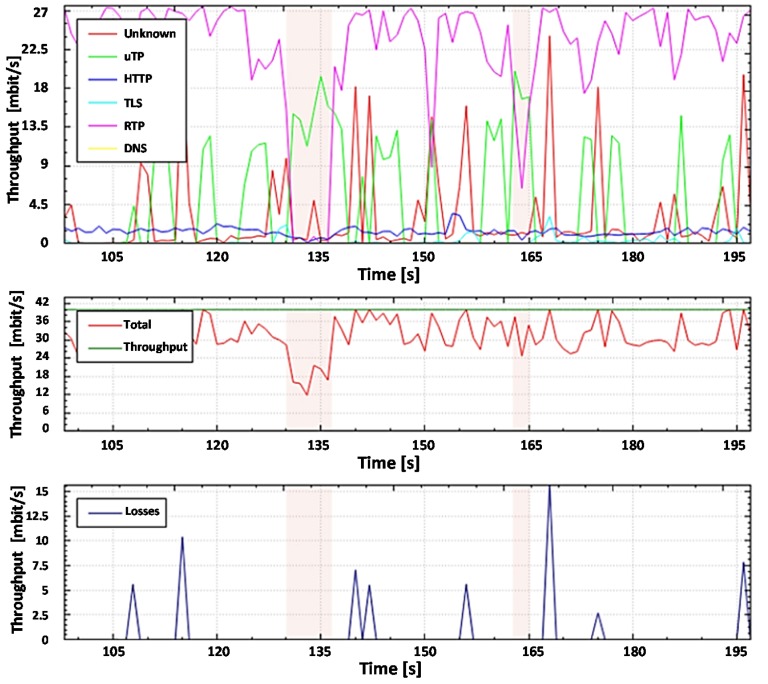
Throughput monitoring, in the transmission of legitimate and non-legitimate traffic (Non-Spoofed UDP Floods attack) using the proposed DPI system.

Using the proposed system, in the 3-second monitoring window, the generated attack is detected as a uTP protocol but as a suspicious of anomaly. Since the evaluated Hurst parameter is not within the range *(-1 S_N_;+1 S_N_*) according to the algorithm proposed in Figure 4, restrictive measures (sending a message to the administrator and limiting the throughput for this traffic) will be taken. Since it is not always reasonable to block "malicious" traffic, the operator may refuse to block it completely and replace it with partial blocking. This is achieved by lowering its priority. In this case, data that have a higher priority of the operator is transmitted first (in this work, all data that are not torrents have a higher priority). During the operation of the traffic prioritization mode, "malicious" traffic is transmitted only when it does not degrade the quality of other services. In Figure 26, the red color indicates the areas where the load is insignificant. For the operator, this means a simple channel, so to prevent downtime, these places are "filled" with torrent traffic. In this case, sufficient QoS is provided for high-priority services, but these torrents are not completely blocked. Using this mode, at 52.52 Mbps, the total loss was 2.27% and the maximum current loss was 19%. 

**Figure 27:**
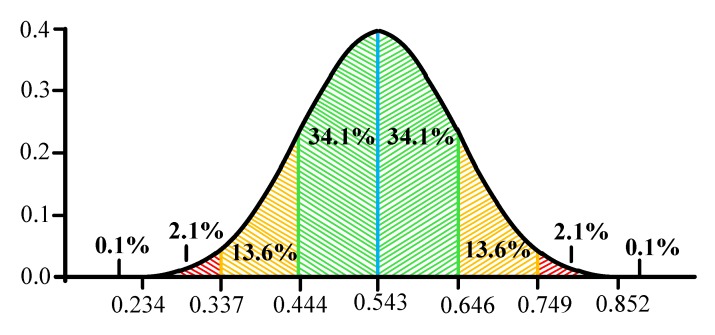
Range of values (-3SN;+3SN) for normal UDP traffic for monitoring windows = 15 seconds.

However, according to the proposed anomaly detection algorithm after the 3-second monitoring window, the system will wait for the value of the 15-second monitoring window and compare the Hurst parameter with the tables calculated in the training phase. The range of values *(-3S_N_;+3S_N_)* for the normal UDP traffic for monitoring windows = 15 seconds is depicted in Figure 27.

**Figure 28:**
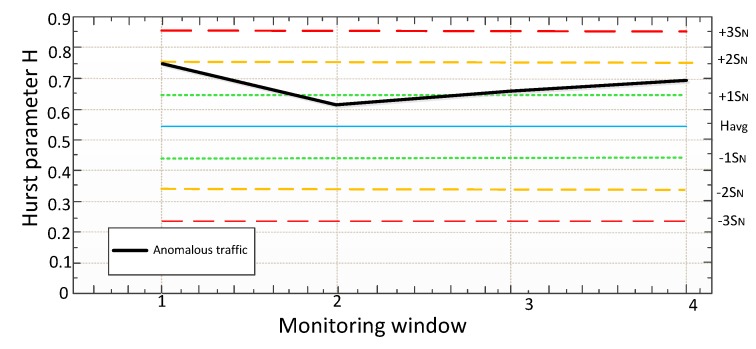
Values in 60 seconds with a 15-second monitoring window for the "anomaly" class (Non-Spoofed UDP Floods attack).

The evaluated Hurst parameter for monitoring windows = 15 seconds is within the range *(+1S_N_;+2S_N_).* According to the algorithm, if the deviation range lies in (+1S_N_; +2 S_N_), restrictive measures will be taken, and the system will continue to wait for the minute window values. Plotting for the minute monitoring window makes no sense, because the value exists in a single copy and a simple comparison of H values of the H_avg1-60_ ⩽ H will be used as a criterion of anomalies. It can also be seen from Table 3 and Table 4 that for this experiment, H_avg1-60_ = 0.403 and H = 0.599, at which it is possible to consider that the traffic is anomalous, relative to the reference values 0.403 < 0.599. Then, the system automatically blocked the anomaly traffic, which allowed releasing the system throughput and improving quality of service parameters, namely to reduce losses. Figure 28 shows for which of the monitoring windows the H values of anomalous traffic exceeded the threshold and for which range they fell in using the proposed algorithm (see Figure 4).

**Figure 29:**
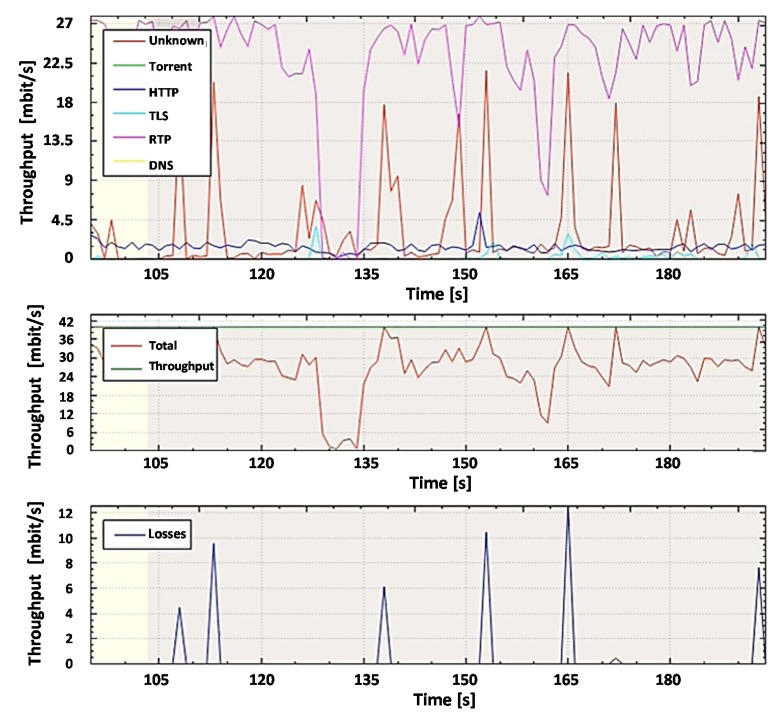
Throughput monitoring, in the transmission of legitimate and non-legitimate traffic (Non-Spoofed UDP Floods attack) using the proposed DPI system after anomaly blocking.

Throughput monitoring, in the transmission of legitimate and non-legitimate traffic (Non-Spoofed UDP Floods attack) using the proposed DPI system after anomaly blocking is depicted in Figure 29. After blocking the anomaly traffic, the maximum current loss is 16% and the level of total loss, at a total interface input load of 53.736 Mbit/s, decreased in ID 7.2% to 2% compared to the SolarWinds DPI system.

Based on the results of the method testing on real network traffic, we can conclude that the ideas proposed by the authors work well. The method of calculation of reference values presented in Section 3 has partially confirmed the ability to reflect the traffic state. It can also be seen from the data obtained in the course of testing (graphs and tables) that the anomaly quite clearly stands out against the conditionally normal graph.

## 6. Discussion

The proposed method uses uncontrolled detection techniques. It is worth considering the nature of detection systems that use uncontrolled detection techniques (when the system knows nothing about the norm and anomalies before starting).

This leads to the conclusion that there is a high probability of false alarms; however, this is typical for all behavioral intrusion detection systems. The situation could be corrected by further improvement of the method and shifting toward a semi-controlled detection technique where the values for the "norm" class are known in advance. However, this entails more preparatory work at the stage of practical implementation of the system based on the proposed method.

The proposed system has knowledge of the ”norm” class by most of the existing protocols, such as DNS, HTTP, RTP, Torrent, and TLS. The development software DPI system can detect attacks such as SYN Flood, HTTP Fragmentation, UDP Flood, DNS Flood, Media Data Flood, and Non-Spoofed UDP Flood. The proposed DPI system has been tested and implemented in the corporate network Lviv Polytechnic National University infrastructure. It allowed to configure the first line of defense against network attacks, taking into account the identified incidents and sources of threats not previously considered in the standard protection means, which increases the speed of response to emerging threats and the level of cyber security of the organization as a whole. By installing the DPI systems at key points in the network, network administrators are able to detect and restrict employees who consume large amounts of personal traffic and users who violate corporate network and Internet access policies. This level of monitoring enables the efficient management of traffic prioritization, providing load reduction and increased channel availability, and ensuring the reliable operation of critical services. Depending on the tasks, the proposed DPI system allows to solve problems related to:

- collection and processing of statistics on network load, providing administrators with detailed information on channel utilization;
- dynamic traffic prioritization and bandwidth management for specific applications, providing optimized channel utilization;
- network traffic management, using the capabilities of DPI solutions to redirect selected traffic to other traffic-handling devices;
- scan traffic for viruses and network anomaly;

The limitation of the proposed solution is that for the effective functioning of the method implemented in the DPI system to detect anomalies, it is necessary to know in advance the dataset, which shows the assessment of the Hurst parameter of normal traffic without anomalies.

The proposed software DPI system is a significant innovation in network technology, which forms the basis for many modern and next-generation services.

In the future, we want to expand the proposed DPI system to detect other attacks. However, the implementation of such a system based on the author’s method is beyond the scope of this work and will be the subject of further research.

## 7. Conclusions

The main requirement for modern Deep Packet Inspection (DPI) systems is the ability to detect anomalies in information processes to identify unknown types of attacks. We have reviewed the existing methods and software products for analyzing network anomalies. We considered the main disadvantages of existing anomaly detection methods, such as a high level of false positives and omissions of cyber attacks, weak capabilities to detect new attacks, the lack of ability to detect an attack in its initial stages, the difficulty of detecting intrusions in real time, and significant loading of the system due to complex calculations.

The statistical research of network traffic shows the presence of self-similarity properties, as well as the variability of these characteristics when anomalies occur in the network, which allows using fractal analysis methods to detect attacks. We proposed a method of forming a set of informative features formalizing normal and anomalous behavior of the network traffic on the basis of evaluating the Hurst (H) parameter. A rescaled range (RS) method to evaluate the Hurst parameter has been chosen. In spite of the fact that RS analysis gives only an approximate value of the Hurst index, the decisive factor was the simplicity of calculations. 

Schemes describing the detection algorithm are presented, as well as a detailed description of the logical components and stages of the anomaly detection system operation. System actions at the detection step are described in detail. An example of a table of subscriber traffic values is given.

A new software DPI system for network traffic analysis and anomaly detection based on Hurst parameter estimation has been proposed. We compared the proposed software DPI system with existing SolarWinds DPI for the possibility of network traffic anomaly detection and prevention. A test bed for simulating anomalous activity and capturing data is described. As a result of the experiment, we proved that the existing system was unable to detect non-legitimate traffic (Non-Spoofed UDP Floods attack). The system detected this anomaly as legitimate uTP traffic. As a result, the attack led to quality-of-service degradation (the total loss rate rose to 7.2%, while the maximum percentage of current loss was 48%) of legitimate traffic due to the significant use of throughput. Unlike the existing high cost of SolarWinds DPI, the proposed software system detected this anomaly as an attack and applied the necessary actions. Namely, it was found that by automatically blocking the detected malicious traffic, the loss rate was reduced by 5% of the total compared to the SolarWinds DPI system. 

The proposed solutions may be ideal for analyzing traffic in backbone networks for security and the detection of attacks. They can be ideal for protecting critical data and maintaining the continuity of Internet services, including the IoT and WSN communication infrastructure.

## Figures and Tables

**Table 1:** Example of a subscriber table.

IP.addr = XX.XX.XX.XX			Another.data		
Int., s.	H_1_	H_2_	H_3_	H_4_	H_n_
Window.size = 3 sec.					
1–3	H_1–3_1	H_1–3_2	H_1–3_3	H_1–3_4	H_1–3_n
4–6	H_4–6_1	H_4–6_2	H_4–6_3	H_4–6_4	H_4–6_n
7–9	H_7–9_1	H_7–9_2	H_7–9_3	H_7–9_4	H_7–9_n
…–…	…	…	…	…	…
58–60	H_58–60_1	H_58–60_2	H_58–60_3	H_58–60_4	H_58–60_n
H_avg(3sec)_	H_avg_1	H_avg_2	H_avg_3	H_avg_ 4	H_avg_ n
S_N__(3sec)_	S_N__(3sec)_1	S_N__(3sec)_1	S_N__(3sec)_1	S_N__(3sec)_1	S_N__(3sec)_1
Window.size = 15 sec.					
1–15	H_1–15_1	H_1–15_2	H_1–15_3	H_1–15_4	H_1–15_n
16–30	H_16–30_1	H_16–30_2	H_16–30_3	H_16–30_4	H_16–30_n
31–45	H_31–45_1	H_31–45_2	H_31–45_3	H_31–45_4	H_31–45_n
46–60	H_46–60_1	H_46–60_2	H_46–60_3	H_46–60_4	H_46–60_n
H_avg(15sec)_	H_avg_1	H_avg_2	H_avg_3	H_avg_ 4	H_avg_ n
S_N__(15sec)_	S_N__(15sec)_1	S_N__(15sec)_1	S_N__(15sec)_1	S_N__(15sec)_1	S_N__(15sec)_1
Window.size = 60 sec.					
1–60	H_1–3_1	H_1–3_2	H_1–3_3	H_1–3_4	H_1–3_n
H_avg(60sec)_	H_avg_ 1	H_avg_2	H_avg_ 3	H_avg_ 4	H_avg_ n

**Table 2:** Subscriber tables for TCP packets of web traffic.

Int., s. = 3	H_3_	Int., s. = 15	H_15_	Int., s. = 60	H_60_
1–3	1.35	1–15	0.4	1–60	0.5
4–6	1.35				
7–9	1.33				
10–12	1.7				
13–15	1.7				
16–18	1.65	16–30	0.6		
19–21	1.35				
22–24	1.35				
25–27	–				
28–30	–				
31–33	1.35	31–45	0.62		
34–36					
37–39					
40–42					
43–45	1.7				
46–48	1.35	46–60	0.67		
49–51					
52–54					
55–57					
58–60					
H_avg(3sec)_	1.47	H_avg (15sec)_	0.57	H_avg (60sec)_	0.5
S_N(3sec)_	0.16	S_N (15sec)_	0.19	–	–

**Table 3:** Tables of subscribers for UDP packets for "normal" class.

Int., s. = 3	H_3_	Int., s. = 15	H_15_	Int., s. = 60	H_60_
1–3	1.355	1–15	0.631	1–60	0.403
4–6	1.355				
7–9	–				
10–12	–				
13–15	–				
16–18	1.611	16–30	0.470		
19–21	1.611				
22–24	1.611				
25–27	1.355				
28–30	1.355	31–45	0.631	1–60	0.403
31–33	1.355	31–45	0.631		
34–36	–				
37–39	–				
40–42	–				
43–45	1.355				
46–48	–	46–60	0.438		
49–51	–				
52–54	1.355				
55–57	–				
58–60	1.355				
H_avg(3sec)_	1.425	H_avg (15sec)_	0.543	H_avg (60sec)_	0.403
S_N__(3sec)_	0.120	S_N__(15sec)_	0.103	–	–

**Table 4:** Tables of subscriber for UDP packets for the "anomaly" class (Non-Spoofed UDP Floods attack).

Int., s. = 3	H_3_	Int., s. = 15	H_15_	Int., s. = 60	H_60_
1–3	1.681	1–15	0.745	1–60	0.599
4–6	1.709				
7–9	1.489				
10–12	1.705				
13–15	1.437	1–15	0.745	1–60	0.599
16–18	1.539	16–30	0.613	1–60	0.599
19–21	1.706				
22–24	1.526				
25–27	1.708				
28–30	1.607				
31–33	1.691	31–45	0.659		
34–36	1.392				
37–39	1.630				
40–42	1.596				
43–45	1.507				
46–48	1.592	46–60	0.694		
49–51	1.703				
52–54	1.682				
55–57	1.547				
58–60	1.647				
